# Supplementary figures and images for: The AAA-ATPase Yta4/ATAD1 interacts with the mitochondrial divisome to inhibit mitochondrial fission
Source: PLoS Biol. 2023 Aug 17;21(8):e3002247. doi: 10.1371/journal.pbio.3002247 (PMC10465003; doi:10.1371/journal.pbio.3002247)

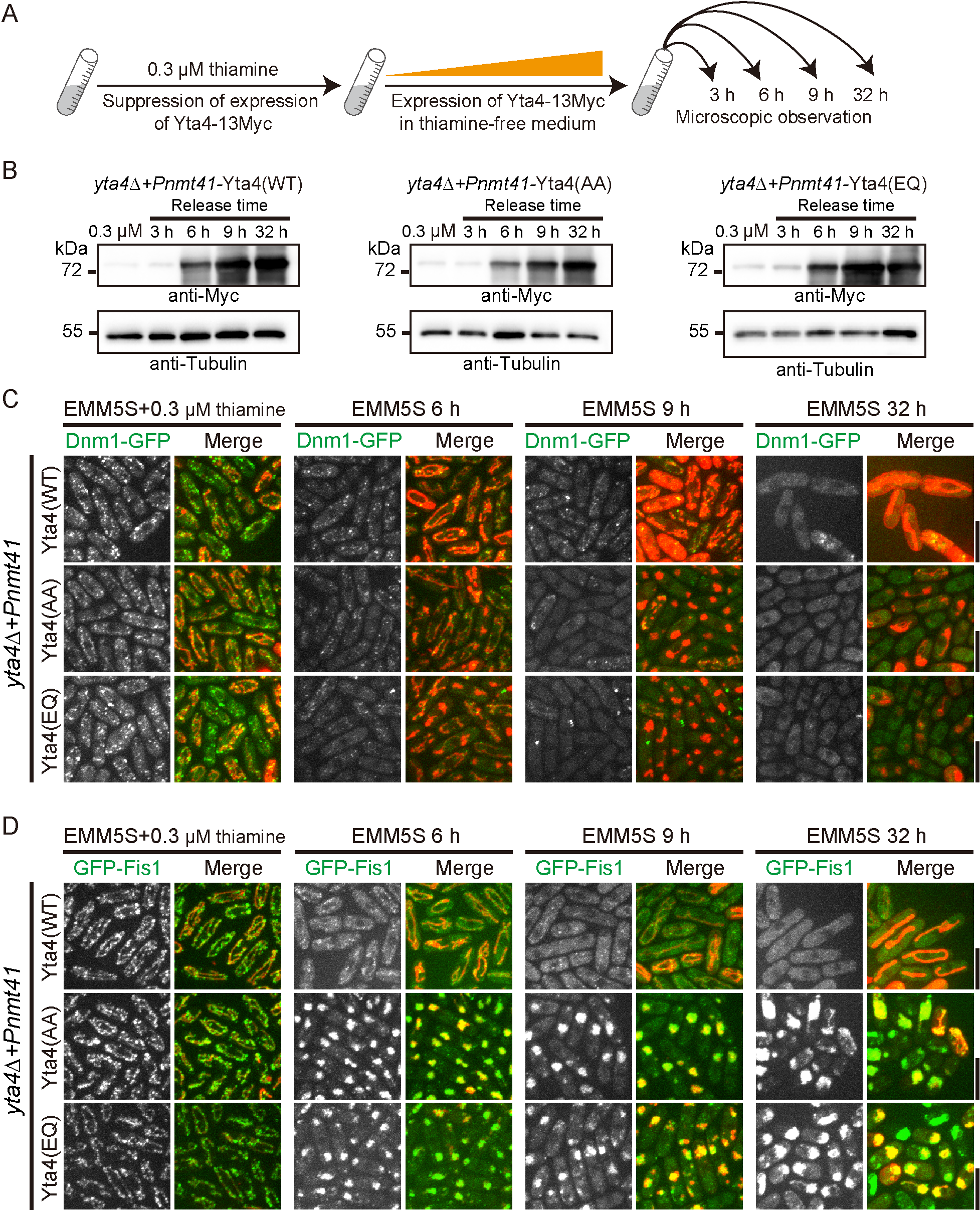

Supplement: S1 Fig — (A) Diagram illustrating the experimental procedure. Briefly, cells were precultured in EMM5S medium containing 0.3 μM thiamine, a chemical used to suppress the promoter nmt41; cells at the exponential phase were collected, washed, and cultured in thiamine-free EMM5S medium to allow expression of Yta4-13Myc (indicated by the yellow triangle) from the nmt41 promoter. After culture in the thiamine-free EMM5S medium for 3, 6, 9, and 32 h, the cells were collected for microscopic observation and analysis by western blotting. (B) Testing the expression of Yta4(WT)/(AA)/(EQ)-13Myc (ectopically expressed from the nmt41 promoter) in yta4∆ cells cultured in EMM5S medium containing 0.3 μM thiamine or thiamine-free EMM5S medium for the indicated time. Western blotting was performed with antibodies against Myc and Tubulin. (C) Maximum projection images of Dnm1-GFP-expressing yta4Δ cells that carry the indicated variants of Yta4-13Myc (from the nmt41 promoter). Cells were cultured in EMM5S medium containing 0.3 μM thiamine or thiamine-free EMM5S medium for the indicated time, and mitochondria were stained with MitoTracker Red. Scale bars, 10 μm. (D) Maximum projection images of GFP-Fis1-expressing yta4Δ cells that carry the indicated variants of Yta4-13Myc (from the nmt41 promoter). Cells were cultured in EMM5S medium containing 0.3 μM thiamine or thiamine-free EMM5S medium for the indicated time, and mitochondria were stained with MitoTracker Red. Scale bars, 10 μm. (TIF) [file pbio.3002247.s001.tif]

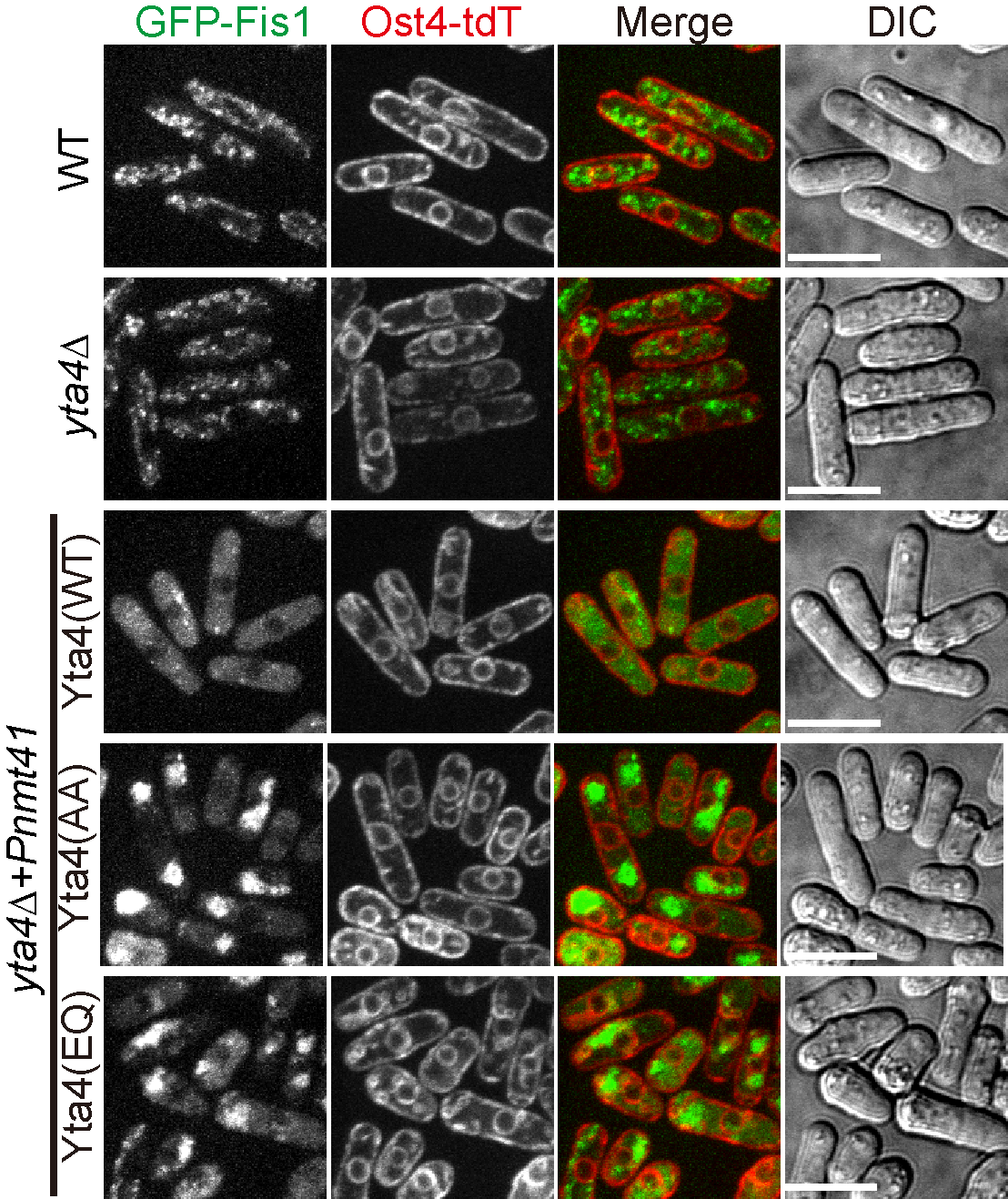

Supplement: S2 Fig — Maximum projection images of the indicated cells expressing GFP-Fis1 and Ost4-tdTomato (an ER marker). Cells were cultured in thiamine-free EMM5S medium for 20 h. Note that mitochondria were aggregated in cells expressing Yta4(AA) and Yta4(EQ), as shown in S1C and S1D Fig. Overexpression of Yta4 caused delocalization of GFP-Fis1 from mitochondria, and delocalized GFP-Fis1 likely localized within the cytoplasm, which did not colocalized with the ER marked by Ost4-tdTomato. Scale bars, 10 μm. (TIF) [file pbio.3002247.s002.tif]

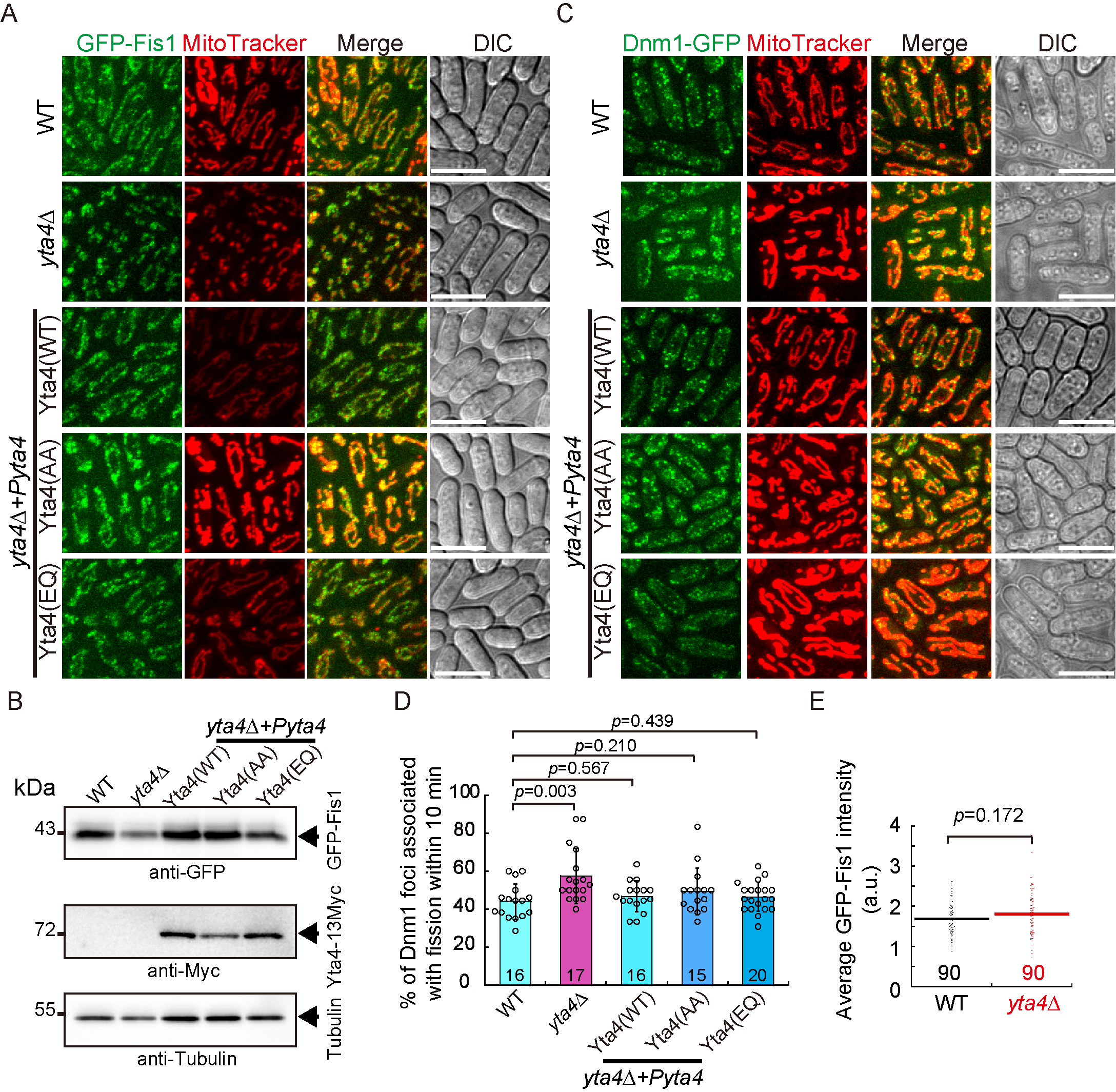

Supplement: S3 Fig — (A) Maximum projection images of the indicated cells expressing GFP-Fis1 and Yta4(WT)/Yta4(AA)/Yta4(EQ)-13Myc (from the yta4 promoter). Mitochondria were stained with MitoTracker Red. Note that the absence of Yta4 caused mitochondrial fragmentation and the expression of Yta4(WT)/Yta4(AA)/Yta4(EQ)-13Myc rescued the mitochondrial phenotype caused by the absence of Yta4. Scale bars, 10 μm. (B) Testing the expression of GFP-Fis1 and different Yta4-13Myc variants (expressed ectopically at the endogenous level) in the indicated cells used in (A) by western blotting with antibodies against GFP, Myc, and Tubulin. Note that the expression levels of Yta4(WT)-13Myc and Yta4(EQ)-13Myc were comparable but the expression level of Yta4(AA)-13Myc was relatively less. (C) Maximum projection images of the indicated cells expressing Dnm1-GFP and Yta4(WT)/Yta4(AA)/Yta4(EQ)-13Myc (from the yta4 promoter). Mitochondria were stained with MitoTracker Red. Note that the absence of Yta4 consistently caused mitochondrial fragmentation and the expression of Yta4(WT)/ Yta4(AA)/Yta4(EQ)-13Myc rescued the mitochondrial phenotype caused by the absence of Yta4. Scale bar, 10 μm. (D) Quantification of the percentage of Dnm1-associated mitochondrial fission. Statistical analysis was performed by Student’s t test. The top of the column indicates the mean while bars indicate SD. The number of cells analyzed is shown on the x-axis. Experiments were repeated twice, and raw data are available in S1 Data. (E) Quantification of the average intensity of GFP-Fis1 signals on mitochondria in WT and yta4Δ cells. Cell number is indicated, and a.u. means arbitrary unit. Statistical analysis was performed by the Wilcoxon–Mann–Whitney rank sum test. Raw data are available in S1 Data. (TIF) [file pbio.3002247.s003.tif]

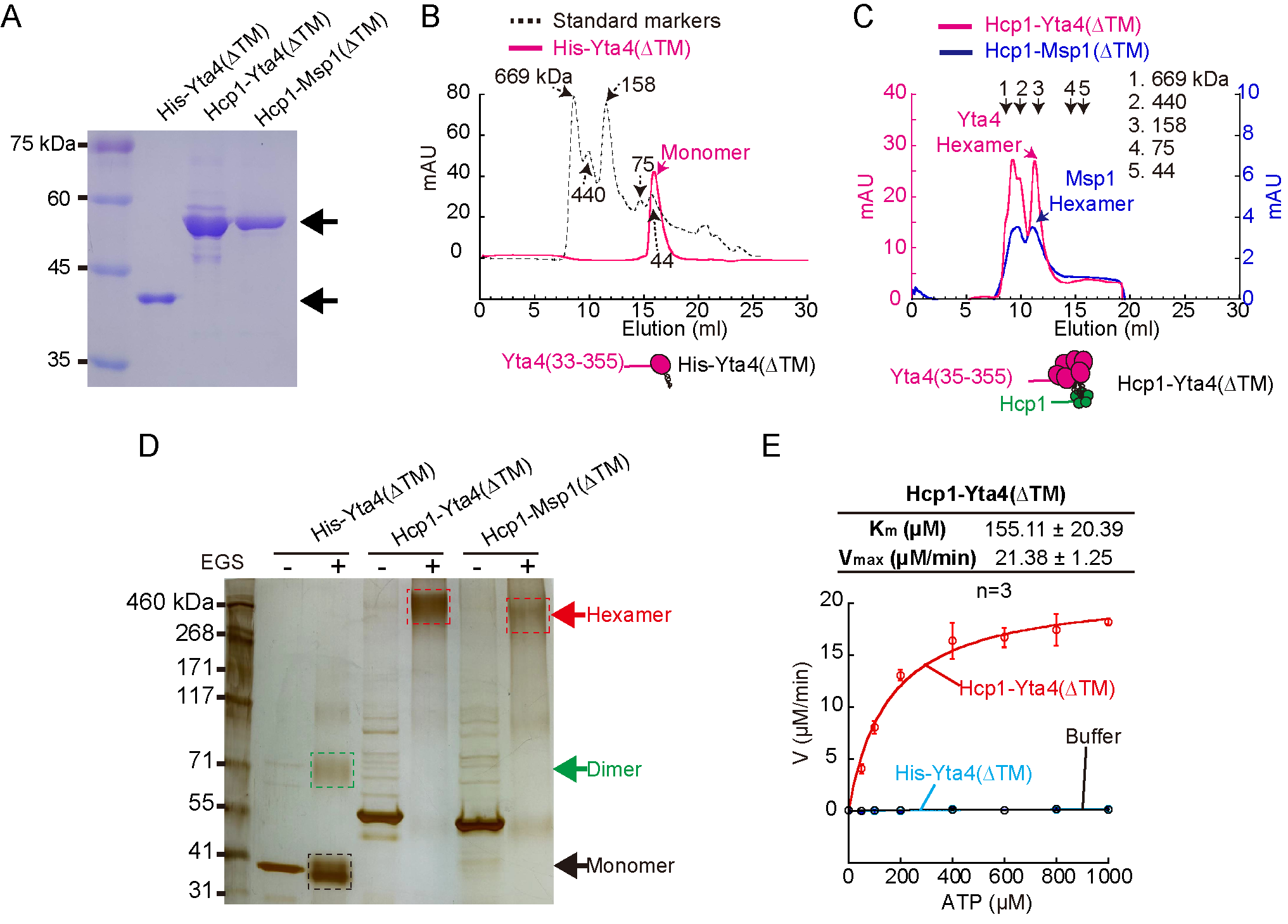

Supplement: S4 Fig — (A) The recombinant proteins His-Yta4(∆TM), Hcp1-Yta4(∆TM), and Hcp1-Msp1(∆TM) used in the analysis of size-exclusion chromatography (shown in B and C). (B and C) Size-exclusion chromatography profiles of the indicated proteins in (A). Protein standards are indicated. Raw data are available in S1 Data. (D) Cross-linking assays. Red, green, and black arrows indicate hexamers, dimers, and monomers, respectively. The proteins were treated with the cross-linking reagent EGS (“+”) or DMSO (“-”). (E) ATPase kinetics assays were performed with the indicated proteins: 1 μM Hcp1-Yta4(∆TM) and 1 μM His-Yta4(∆TM). The buffer was used as a negative control. The initial rate (V) was determined using the slope of generated phosphate measured by colorimetric assays at 37°C at the indicated concentration of ATP. The curves were created by fitting to a Michaelis–Menten model, and Km and Vmax values were obtained from the fitting. Data points are averages, while error bars represent SD (from 3 independent experiments). Values in the table are average±SD. Raw data are available in S1 Data. (TIF) [file pbio.3002247.s004.tif]

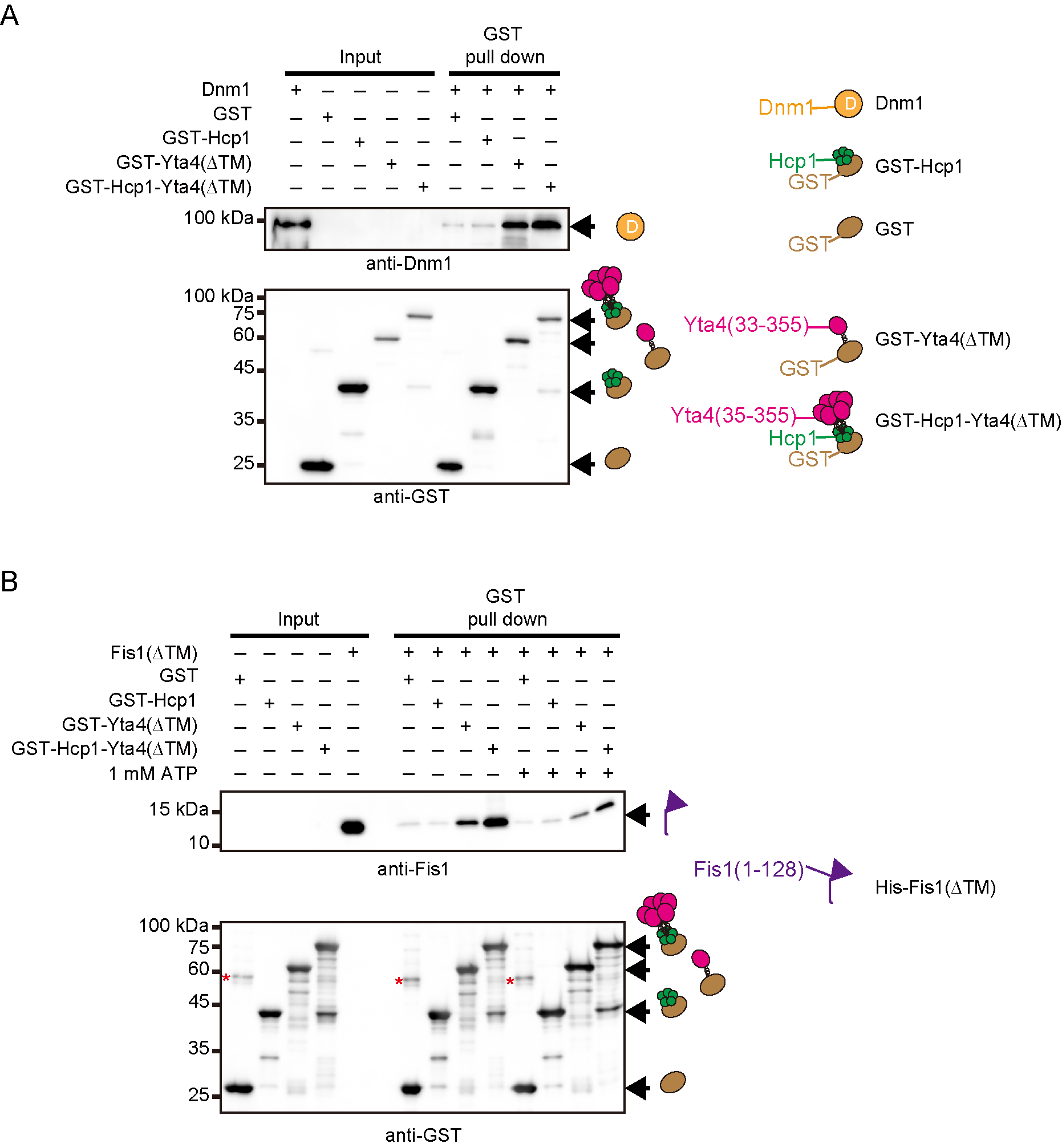

Supplement: S5 Fig — (A) GST pull-down assays were performed to test the interaction between Dnm1 and GST-Hcp1-Yta4(∆TM), GST-Yta4(∆TM), GST-Hcp1, or GST. The GST-fused proteins were indicated by arrows. Western blotting was performed with antibodies against Dnm1 and GST. (B) GST pull-down assays were performed to test the interaction between His-Fis1(∆TM) and GST-Hcp1-Yta4(∆TM), GST-Yta4(∆TM), GST-Hcp1, or GST in the presence or absence of 1 mM ATP. Western blotting was performed with antibodies against Fis1 and GST. (TIF) [file pbio.3002247.s005.tif]

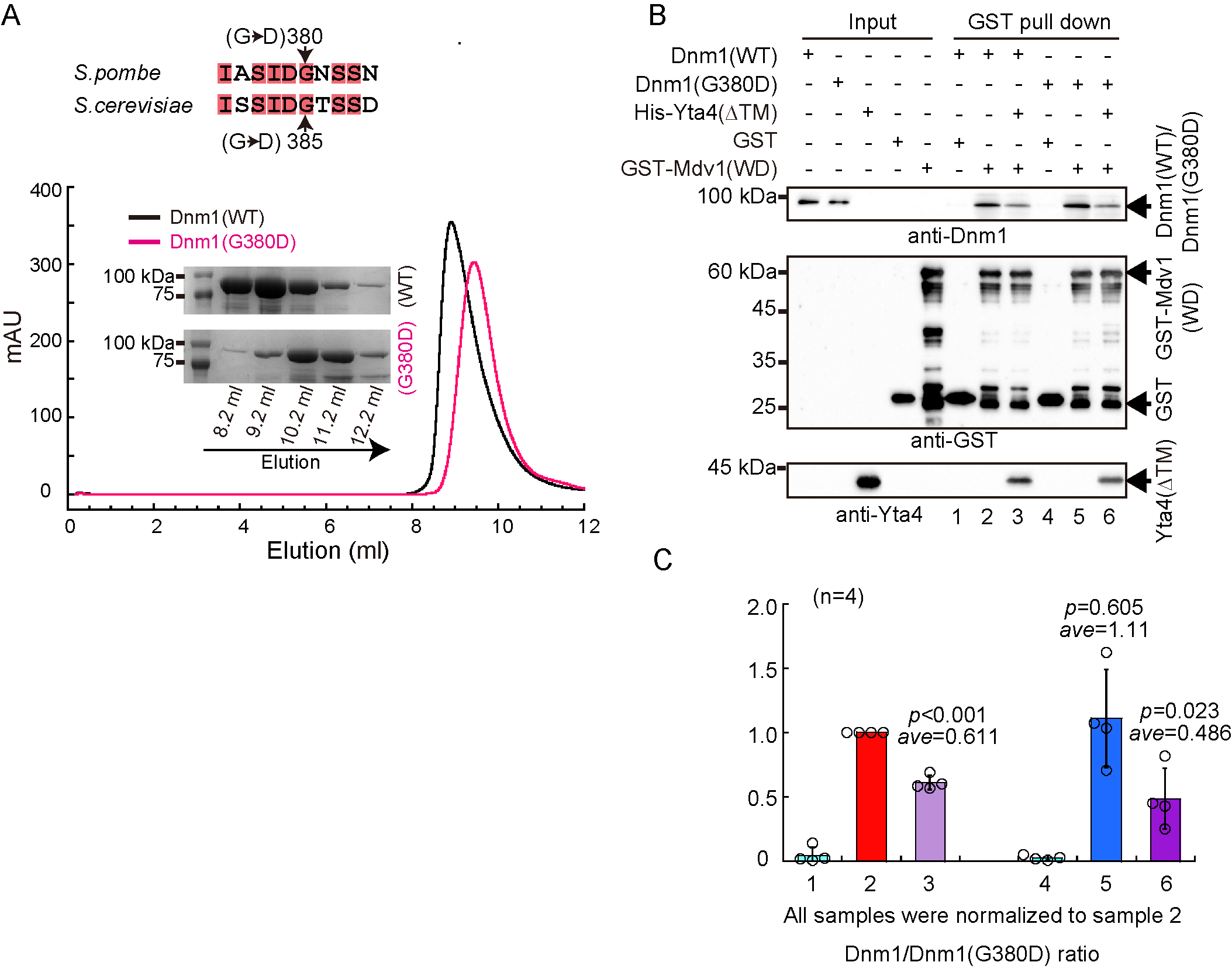

Supplement: S6 Fig — (A) Top: Alignment of the amino acid sequences of Dnm1 and its budding yeast homolog. The conserved residues are highlighted in red, and the residues mutated (G was mutated into D) in Dnm1 are indicated by arrows. Bottom: Size-exclusion chromatography profiles of the indicated proteins. (B) GST pull-down assays were performed to test the interactions between GST-Mdv1(WD) and Dnm1(WT) and Dnm1(G380D) in the presence or absence of His-Yta4(∆TM). GST was used as a negative control. Note that the presence of His-Yta4(∆TM) significantly reduced the precipitate of Dnm1(WT) and Dnm1(G380D) by GST-Mdv1(WD). In addition, the precipitate of Dnm1(WT) and Dnm1(G380D) by GST-Mdv1(WD) was comparable. (C) Quantification of Dnm1 pulled down by GST-fused proteins. The band intensity of Dnm1 was first normalized to the band intensity of corresponding GST proteins, and the ratio of the indicated samples on x-axis of Dnm1 intensity was then calculated by normalizing to sample 2. Four independent experiments were performed. The top of the column represents the mean (indicated by ave), and single group Student’s t test was used to calculate the p values. Raw data are available in S1 Data. (TIF) [file pbio.3002247.s006.tif]

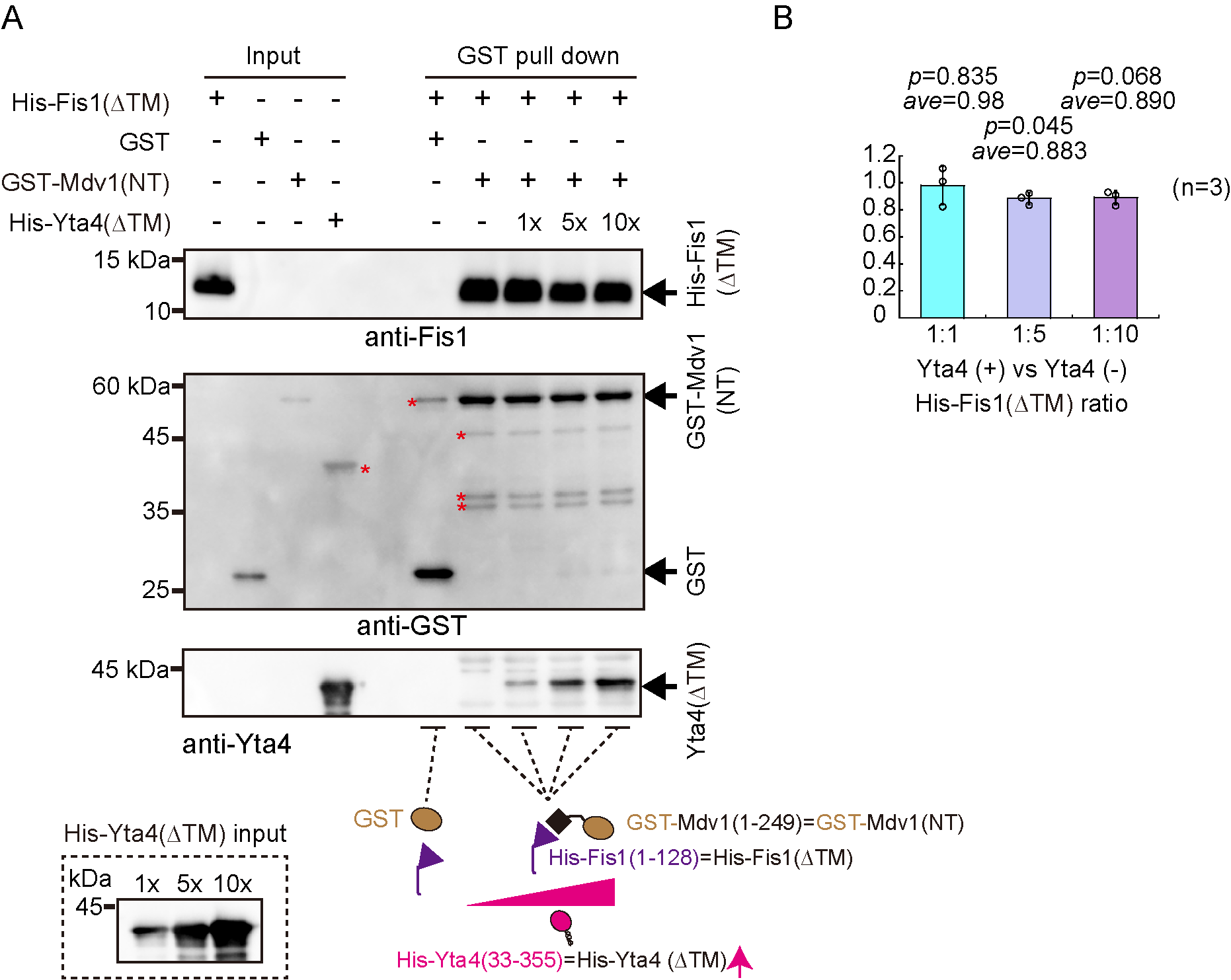

Supplement: S7 Fig — (A) GST pull-down assays were performed to test the interaction between GST-Mdv1(NT) and His-Fis1(∆TM) in the presence or absence of His-Yta4(∆TM). Different concentration ratios (i.e., 1:1, 1:5, and 1:10) of Mdv1 to Yta4 were tested (see the input of His-Yta4(∆TM) at the bottom left of the graph). GST was used as a negative control. Note that the presence of His-Yta4(∆TM) did not significantly affect the interaction between GST-Mdv1(NT) and His-Fis1(∆TM). (B) Quantification of His-Fis1(∆TM) pulled down by GST-fused proteins. The band intensity of His-Fis1(∆TM) was first normalized to the band intensity of corresponding GST proteins, and the ratio of the normalized values of His-Fis1(∆TM) intensity was then calculated. Yta4(-) and Yta4(+) indicate the samples, in which GST-Mdv1(NT) plus His-Fis1(∆TM), and GST-Mdv1(NT) plus His-Fis1(∆TM) and His-Yta4(∆TM) were used, respectively. Three independent experiments were performed. The top of the column represents the mean (indicated by ave), and single group Student’s t test was carried out to calculate the p values. Raw data are available in S1 Data. (TIF) [file pbio.3002247.s007.tif]

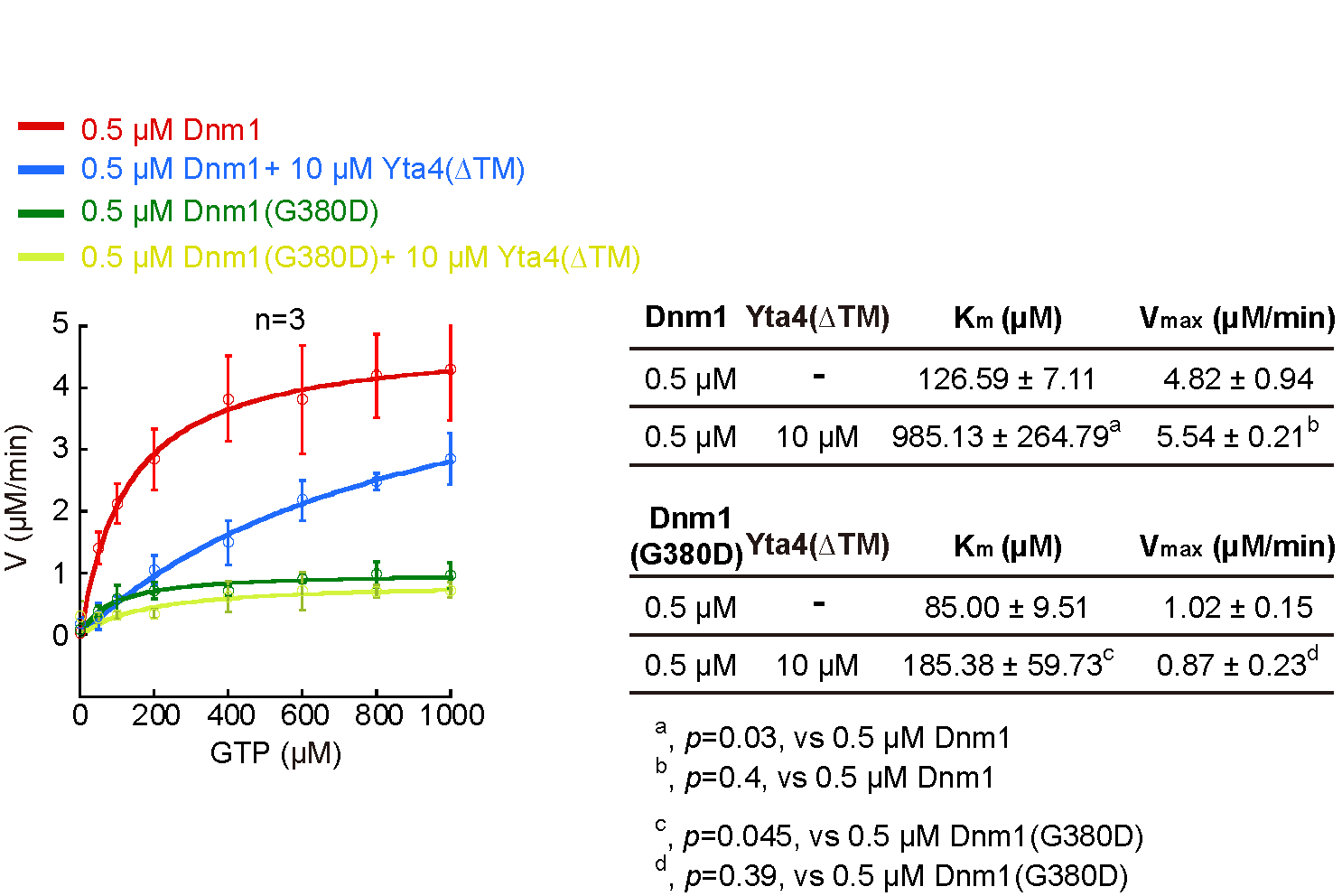

Supplement: S8 Fig — GTPase kinetics assays were performed with the indicated proteins: 0.5 μM Dnm1(WT)/Dnm1(G380D) alone or in the presence of 10 μM His-Yta4(∆TM). The curves were created by fitting to a Michaelis–Menten model, and Km and Vmax values were obtained from the fitting. Data points are averages, while error bars represent SD (from 3 independent experiments). Values in the table are average ± SD. Student’s t test was used to calculate p values. Raw data are available in S1 Data. (TIF) [file pbio.3002247.s008.tif]
