# Supplementary material for: The AAA-ATPase Yta4/ATAD1 interacts with the mitochondrial divisome to inhibit mitochondrial fission
Source: PLoS Biol. 2023 Aug 17;21(8):e3002247. doi: 10.1371/journal.pbio.3002247 (PMC10465003; doi:10.1371/journal.pbio.3002247)
Supplement: S1 Raw Images — (PDF) [file pbio.3002247.s009.pdf]

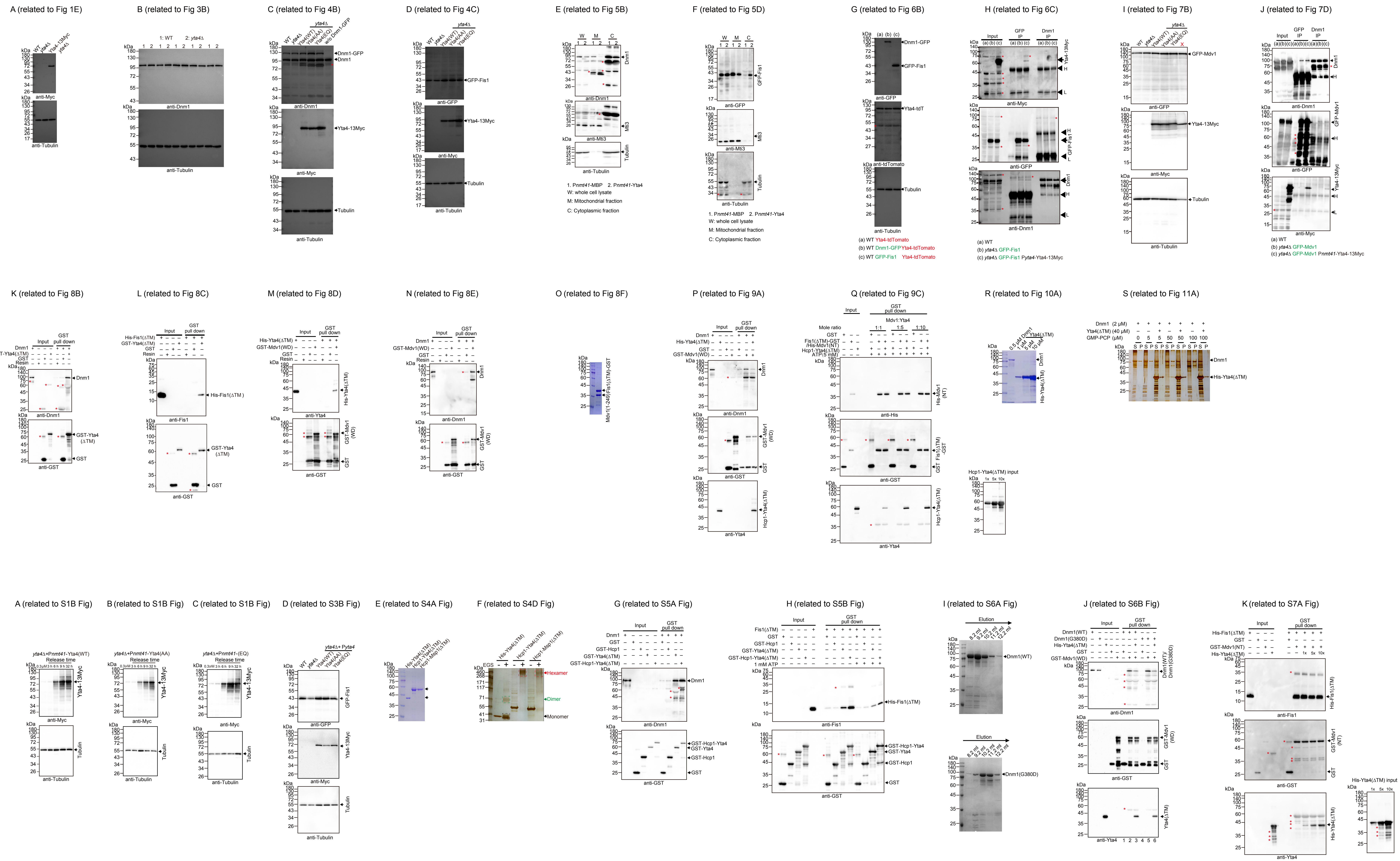
A (related to Fig 1E)
B (related to Fig 3B)
C (related to Fig 4B)
D (related to Fig 4C)
E (related to Fig 5B)
F (related to Fig 5D)
G (related to Fig 6B)
H (related to Fig 6C)
I (related to Fig 7B)
J (related to Fig 7D)
1: WT 2: yta4Δ
1. Pnmt41-MBP 2. Pnmt41-Yta4
W: whole cell lysate
M: Mitochondrial fraction
C: Cytoplasmic fraction
(a) WT Yta4-tdTomato
(b) WT Dnm1-GFP Yta4-tdTomato
(c) WT GFP-Fis1 Yta4-tdTomato
(a) WT
(b) yta4Δ GFP-Fis1
(c) yta4Δ GFP-Fis1 Pyta4-Yta4-13Myc
(a) WT
(b) yta4Δ GFP-Mdv1
(c) yta4Δ GFP-Mdv1 Pnmt41-Yta4-13Myc
anti-Myc
anti-Tubulin
anti-Dnm1
anti-GFP
anti-Mti3
anti-tdTomato
anti-Yta4
anti-GST
anti-Fis1
anti-His
K (related to Fig 8B)
L (related to Fig 8C)
M (related to Fig 8D)
N (related to Fig 8E)
O (related to Fig 8F)
P (related to Fig 9A)
Q (related to Fig 9C)
R (related to Fig 10A)
S (related to Fig 11A)
Hcp1-Yta4(ΔTM) input
A (related to S1B Fig)
B (related to S1B Fig)
C (related to S1B Fig)
D (related to S3B Fig)
E (related to S4A Fig)
F (related to S4D Fig)
G (related to S5A Fig)
H (related to S5B Fig)
I (related to S6A Fig)
J (related to S6B Fig)
K (related to S7A Fig)
yta4Δ+Pnmt41-Yta4(WT)
yta4Δ+Pnmt41-Yta4(AA)
yta4Δ+Pnmt41-(EQ)
Release time
Hexamer
Dimer
Monomer
Elution
His-Yta4(ΔTM) input
