# Supplementary material for: The AAA-ATPase Yta4/ATAD1 interacts with the mitochondrial divisome to inhibit mitochondrial fission
Source: PLoS Biol. 2023 Aug 17;21(8):e3002247. doi: 10.1371/journal.pbio.3002247 (PMC10465003; doi:10.1371/journal.pbio.3002247)
Supplement: S1 Table — (DOCX) [file pbio.3002247.s018.docx]

**S1 Table. Plasmids**

| **Plasmid** | **Genotype** | **Source** |
| --- | --- | --- |
| **Fig 1** |  |  |
| pCF.3132 | pJK210-*Pyta4*-Yta4-13Myc | This study |
| **Fig 3** |  |  |
| pCF.2779 | pJK148-*Pase1*-Dnm1-GFP | This study |
| **Fig 4** |  |  |
| pCF.2779 | pJK148-*Pase1*-Dnm1-GFP | This study |
| pCF.3134 | pJK210-*Pnmt41*-Yta4(WT)-13Myc | This study |
| pCF.3582 | pJK210-*Pnmt41*-Yta4(W165A/F166A)-13Myc | This study |
| pCF.3568 | pJK210-*Pnmt41*-Yta4(E192Q)-13Myc | This study |
| pCF.3344 | pFA6a-KanMX-*Pase1*-GFP | This study |
| **Fig 5** |  |  |
| pCF.2779 | pJK148-*Pase1*-Dnm1-GFP | This study |
| pCF.3134 | pJK210-*Pnmt41*-Yta4(WT)-13Myc | This study |
| pCF.3157 | pJK210-*Pnmt41*-MBP-13Myc | This study |
| pCF.3344 | pFA6a-KanMX-*Pase1*-GFP | This study |
| **Fig 6** |  |  |
| pCF.2779 | pJK148-*Pase1*-Dnm1-GFP | This study |
| pCF.3344 | pFA6a-KanMX-P*ase1*-GFP | This study |
| pCF.3132 | pJK210-*Pyta4*-Yta4(WT)-13Myc | This study |
| pPT.166 | pFA6a-tdTomato-NatMX6 | This study |
| **Fig 7** |  |  |
| pCF.3344 | pFA6a-KanMX-*Pase1*-GFP | This study |
| pCF.3134 | pJK210-*Pnmt41*-Yta4(WT)-13Myc | This study |
| pCF.3582 | pJK210-*Pnmt41*-Yta4(W165A/F166A)-13Myc | This study |
| pCF.3568 | pJK210-*Pnmt41*-Yta4(E192Q)-13Myc | This study |
| pPT.166 | pFA6a-tdTomato-NatMX6 | This study |
| **Fig 8** |  |  |
| pCF.3411 | pET28-His-sumo-Dnm1 | This study |
| pCF.3556 | pGEX-6p-1-GST-Yta4(a.a. 33-355) | This study |
| pCF.3462 | pGEX-6p-1-GST | This study |
| pCF.3676 | pET28-His-Fis1(a.a. 1-128) | This study |
| pCF.4669 | pGEX-6p-1-GST-Mdv1(a.a. 319-651) | This study |
| pCF.4653 | pGEX-6p-1-Fis1(a.a. 1-128)-GST | This study |
| pCF.4665 | pET28-His-Mdv1(a.a. 1-249) | This study |
| pCF.3459 | pET28-His-Yta4(a.a. 33-355) | This study |
| **Fig 9** |  |  |
| pCF.3411 | pET28-His-sumo-Dnm1 | This study |
| pCF.3459 | pET28-His-Yta4(a.a. 33-355) | This study |
| pCF.3462 | pGEX-6p-1-GST | This study |
| pCF.4669 | pGEX-6p-1-GST-Mdv1(a.a. 319-651) | This study |
| pCF.4653 | pGEX-6p-1-Fis1(a.a. 1-128)-GST | This study |
| pCF.4665 | pET28-His-Mdv1(a.a. 1-249) | This study |
| pCF.4622 | pET28-His-sumo-Hcp1-Yta4(a.a. 35-355) | This study |
| **Fig 10** |  |  |
| pCF.3411 | pET28-His-sumo-Dnm1 | This study |
| pCF.3459 | pET28-His-Yta4(a.a. 33-355) | This study |
| **Fig 11** |  |  |
| pCF.3411 | pET28-His-sumo-Dnm1 | This study |
| pCF.3459 | pET28-His-Yta4(a.a. 33-355) | This study |
| **S1 Fig** |  |  |
| pCF.2779 | pJK148-*Pase1*-Dnm1-GFP | This study |
| pCF.3134 | pJK210-*Pnmt41*-Yta4(WT)-13Myc | This study |
| pCF.3582 | pJK210-*Pnmt41*-Yta4(W165A/F166A)-13Myc | This study |
| pCF.3568 | pJK210-*Pnmt41*-Yta4(E192Q)-13Myc | This study |
| pCF.3344 | pFA6a-KanMX-*Pase1*-GFP | This study |
| **S2 Fig** |  |  |
| pCF.3344 | pFA6a-KanMX-*Pase1*-GFP | This study |
| pCF.3134 | pJK210-*Pnmt41*-Yta4(WT)-13Myc | This study |
| pCF.3582 | pJK210-*Pnmt41*-Yta4(W165A/F166A)-13Myc | This study |
| pCF.3568 | pJK210-*Pnmt41*-Yta4(E192Q)-13Myc | This study |
| pPT.166 | pFA6a-tdTomato-NatMX6 | This study |
| **S3 Fig** |  |  |
| pCF.2779 | pJK148-*Pase1*-Dnm1-GFP | This study |
| pCF.3132 | pJK210-*Pyta4*-Yta4(WT)-13Myc | This study |
| pCF.3153 | pJK210-*Pyta4*-Yta4(W165A/F166A)-13Myc | This study |
| pCF.3154 | pJK210-*Pyta4-*Yta4(E192Q)-13Myc | This study |
| pCF.3344 | pFA6a-KanMX-*Pase1*-GFP | This study |
| **S4 Fig** |  |  |
| pCF.3459 | pET28-His-Yta4(a.a. 33-355) | This study |
| pCF.4622 | pET28-His-sumo-Hcp1-Yta4(a.a. 35-355) | This study |
| pCF.4639 | pET28-His-sumo-Hcp1-Msp1 (a.a. 36-362) | This study |
| **S5 Fig** |  |  |
| pCF.3411 | pET28-His-sumo-Dnm1 | This study |
| pCF.3556 | pGEX-6p-1-GST-Yta4(a.a. 33-355) | This study |
| pCF.3462 | pGEX-6p-1-GST | This study |
| pCF.4681 | pGEX-6p-1-GST-Hcp1 | This study |
| pCF.4682 | pGEX-6p-1-GST-Hcp1-Yta4(a.a. 35-355) | This study |
| pCF.3676 | pET28-His-Fis1(a.a. 1-128) | This study |
| **S6 Fig** |  |  |
| pCF.3411 | pET28-His-sumo-Dnm1 | This study |
| pCF.4883 | pET28-His-sumo-Dnm1(G380D) | This study |
| pCF.3459 | pET28-His-Yta4(a.a. 33-355) | This study |
| pCF.3462 | pGEX-6p-1-GST | This study |
| pCF.4669 | pGEX-6p-1-GST-Mdv1(a.a. 319-651) | This study |
| **S7 Fig** |  |  |
| pCF.3676 | pET28-His-Fis1(a.a. 1-128) | This study |
| pCF.3462 | pGEX-6p-1-GST | This study |
| pCF.4768 | pGEX-6p-1-GST-Mdv1(a.a. 1-249) | This study |
| pCF.3459 | pET28-His-Yta4(a.a. 33-355) | This study |
| **S8 Fig** |  |  |
| pCF.3411 | pET28-His-sumo-Dnm1 | This study |
| pCF.4883 | pET28-His-sumo-Dnm1(G380D) | This study |
| pCF.3459 | pET28-His-Yta4(a.a. 33-355) | This study |
