# Supplementary material for: The AAA-ATPase Yta4/ATAD1 interacts with the mitochondrial divisome to inhibit mitochondrial fission
Source: PLoS Biol. 2023 Aug 17;21(8):e3002247. doi: 10.1371/journal.pbio.3002247 (PMC10465003; doi:10.1371/journal.pbio.3002247)
Supplement: S2 Table — (DOCX) [file pbio.3002247.s019.docx]

**S2 Table. Yeast strains**

| **Strain** | **Genotype** | **Source** |
| --- | --- | --- |
| **Fig 1** |  |  |
| pT.286 | WT *ade6*-m210 *leu1*-32 *ura4*-D18 h- | This study |
| CF.5806 | *yta4*Δ:KanR *ade6*-m210 *leu1*-32 *ura4*-D18 h- | This study |
| CF.8698 | Sdh2-mCherry:KanR *ade6*-m210 *leu1*-32 *ura4*-D18 h+ | From Du Lab |
| CF.8760 | *yta4*Δ:KanR Sdh2-mCherry:KanR *ade6*-m210 *leu1*-32 *ura4*-D18 h? | This study |
| CF.9819 | *yta4*Δ:KanR Sdh2-mCherry:KanR *Pyta4*-Yta4-13Myc:*ura+* *ade6*-m210 *leu1*-32 h? | This study |
| **Fig 2** |  |  |
| CF.8698 | Sdh2-mCherry:KanR *ade6*-m210 *leu1*-32 *ura4*-D18 h+ | This study |
| CF.8760 | *yta4*Δ:KanR Sdh2-mCherry:KanR *ade6*-m210 *leu1*-32 *ura4*-D18 h? | This study |
| **Fig 3** |  |  |
| CF.8698 | Sdh2-mCherry:KanR *ade6*-m210 *leu1*-32 *ura4*-D18 h+ | From Du Lab |
| CF.8760 | *yta4Δ*:KanR Sdh2-mCherry:KanR *ade6*-m210 *leu1*-32 *ura4*-D18 h? | This study |
| CF.9790 | *dnm1*Δ:KanR Sdh2-mCherry:KanR *ade6*-m210 *leu1*-32 *ura4*-D18 h- | This study |
| CF.9461 | *dnm1*Δ:KanR *yta4Δ*:KanR Sdh2-mCherry:KanR *ade6*-m210 *leu1*-32 *ura4*-D18 h- | This study |
| CF.8719 | Sdh2-mCherry:KanR *Pase1*-Dnm1-GFP:leu+ *ade6*-m210 *ura4*-D18 h+ | This study |
| CF.9455 | *yta4*Δ:KanR Sdh2-mCherry:KanR *Pase1*-Dnm1-GFP:leu+ *ade6*-m210 *ura4*-D18 h- | This study |
| pT.286 | WT *ade6*-m210 *leu1*-32 *ura4*-D18 h- | This study |
| CF.5806 | *yta4*Δ:KanR *ade6*-m210 *leu1*-32 *ura4*-D18 h- | This study |
| **Fig 4** |  |  |
| CF.8648 | *Pase1*-Dnm1-GFP:*leu+* *ade6*-m210 *ura4*-294-h? | This study |
| CF.9218 | *yta4*Δ:KanR *Pase1*-Dnm1-GFP:*leu+* *ade6*-m210 *ura4*-294-h? | This study |
| CF.9397 | *yta4*Δ:KanR *Pase1*-Dnm1-GFP:*leu+* *Pnmt41*-Yta4(WT)-13Myc:*ura+* *ade6*-m210 h? | This study |
| CF.9398 | *yta4*Δ:KanR *Pase1*-Dnm1-GFP:*leu+* *Pnmt41*-Yta4(W165A/F166A)-13Myc:*ura+* *ade6*-m210 h? | This study |
| CF.9399 | *yta4*Δ:KanR *Pase1*-Dnm1-GFP:*leu+* *Pnmt41*-Yta4(E192Q)-13Myc:*ura+* *ade6*-m210 h? | This study |
| CF.9210 | *Pase1*-GFP-Fis1:KanR *ade6*-m210 *leu1*-32 *ura*-294- h- | This study |
| CF.9380 | *yta4*Δ:KanR *Pase1*-GFP-Fis1:KanR *ade6*-m210 *leu1*-32 *ura4*-294- h+ | This study |
| CF.9400 | *yta4*Δ:KanR *Pase1*-GFP-Fis1:KanR *Pnmt41*-Yta4(WT)-13Myc:*ura+* *ade6*-m210 *leu1*-32 h+ | This study |
| CF.9401 | *yta4*Δ:KanR *Pase1*-GFP-Fis1:KanR *Pnmt41*-Yta4(W165A/F166A)-13Myc:*ura+* *ade6*-m210 *leu1*-32 h+ | This study |
| CF.9417 | *yta4*Δ:KanR *Pase1*-GFP-Fis1:KanR *Pnmt41*-Yta4(E192Q)-13Myc:*ura+* *ade6*-m210 *leu1*-32 h+ | This study |
| **Fig 5** |  |  |
| CF.8909 | *Pase1*-Dnm1-GFP:*leu+ Pnmt41*-MBP-13Myc:*ura+* *ade6*-m210 h+ | This study |
| CF.8650 | *Pase1*-Dnm1-GFP:*leu+* *Pnmt41*-Yta4(WT)-13Myc:*ura+* *ade6*-m210 h+ | This study |
| CF.9212 | *Pase1*-GFP-Fis1:KanR *Pnmt41*-MBP-13Myc:*ura+* *ade6*-m210 *leu1*-32 h- | This study |
| CF.9213 | *Pase1*-GFP-Fis1:KanR *Pnmt41*-Yta4(WT)-13Myc:*ura+* *ade6*-m210 *leu1*-32 h- | This study |
| CF.8569 | *Pnmt41*-MBP-13Myc:*ura+* *ade6*-m210 *leu1*-32 h- | This study |
| CF.7470 | *Pnmt41*-Yta4(WT)-13Myc:*ura+* *ade6*-m210 *leu1*-32 h- | This study |
| **Fig 6** |  |  |
| CF.9817 | Yta4-tdTomato:NatR *ade6*-m210 *leu1*-32 ura4-D18 h+ | This study |
| CF.9818 | Yta4-tdTomato:NatR P*ase1*-Dnm1-GFP:*leu+* *ade6*-m210 *ura4*-294 h- | This study |
| CF.10011 | Yta4-tdTomato:NatR *Pase1*-GFP-Fis1:KanR *ade6*-m210 *leu1*-32 *ura4*? h? | This study |
| pT.286 | WT *ade6*-m210 *leu1*-32 *ura4*-D18 h- | This study |
| CF.9380 | *yta4*Δ:KanR *Pase1*-GFP-Fis1:KanR *ade6*-m210 *leu1*-32 *ura4*-294- h+ | This study |
| CF.9849 | *yta4*Δ:KanR *Pase1*-GFP-Fis1:KanR P*yta4*-Yta4(WT)-13Myc:*ura+* *ade6*-m210 *leu1*-32 h+ | This study |
| **Fig 7** |  |  |
| CF.12945 | *Pase1*-GFP-Mdv1:KanR *ade6*-m210 *leu1*-32 *ura*-294- h- | This study |
| CF.12959 | *yta4*Δ:KanR *Pase1*-GFP-Mdv1:KanR *ade6*-m210 *leu1*-32 *ura*-294- h- | This study |
| CF.13346 | *yta4*Δ:KanR *Pase1*-GFP-Mdv1:KanR *Pnmt41*-Yta4(WT)-13Myc:*ura+* *ade6*-m210 *leu1*-32 h? | This study |
| CF.13222 | *yta4*Δ:KanR *Pase1*-GFP-Mdv1:KanR *Pnmt41*-Yta4(W165A/F166A)-13Myc:*ura+* *ade6*-m210 *leu1*-32 h+ | This study |
| CF.13223 | *yta4*Δ:KanR *Pase1*-GFP-Mdv1:KanR *Pnmt41*-Yta4(E192Q)-13Myc:*ura+* *ade6*-m210 *leu1*-32 h+ | This study |
| CF.12949 | Yta4-tdTomato:NatR *Pase1*-GFP-Mdv1:KanR *ade6*-m210 *leu1*-32 *ura4*? h? | This study |
| pT.286 | WT *ade6*-m210 *leu1*-32 *ura4*-D18 h- | This study |
| **S1 Fig** |  |  |
| CF.9397 | *yta4*Δ:KanR *Pase1*-Dnm1-GFP:*leu+* *Pnmt41*-Yta4(WT)-13Myc:*ura+* *ade6*-m210 h? | This study |
| CF.9398 | *yta4*Δ:KanR *Pase1*-Dnm1-GFP:*leu+* *Pnmt41*-Yta4(W165A/F166A)-13Myc:*ura+* *ade6*-m210 h? | This study |
| CF.9399 | *yta4*Δ:KanR *Pase1*-Dnm1-GFP:*leu+* *Pnmt41*-Yta4(E192Q)-13Myc:*ura+* *ade6*-m210 h? | This study |
| CF.9400 | *yta4*Δ:KanR *Pase1*-GFP-Fis1:KanR *Pnmt41*-Yta4(WT)-13Myc:*ura+* *ade6*-m210 *leu1*-32 h+ | This study |
| CF.9401 | *yta4*Δ:KanR *Pase1*-GFP-Fis1:KanR *Pnmt41*-Yta4(W165A/F166A)-13Myc:*ura+* *ade6*-m210 *leu1*-32 h+ | This study |
| CF.9417 | *yta4*Δ:KanR *Pase1*-GFP-Fis1:KanR *Pnmt41*-Yta4(E192Q)-13Myc:*ura+* *ade6*-m210 *leu1*-32 h+ | This study |
| **S2 Fig** |  |  |
| CF.13014 | *Pase1*-GFP-Fis1:KanR Ost4-tdTomato:NatR *ade6*-m210 *leu1*-32 *ura*-294- h? | This study |
| CF.13162 | *yta4*Δ:KanR *Pase1*-GFP-Fis1:KanR Ost4-tdTomato:NatR *ade6*-m210 *leu1*-32 *ura*-294- h? | This study |
| CF.13016 | *yta4*Δ:KanR *Pase1*-GFP-Fis1:KanR Ost4-tdTomato:NatR *Pnmt41*-Yta4(WT)-13Myc:*ura+* *ade6*-m210 *leu1*-32 h? | This study |
| CF.13018 | *yta4*Δ:KanR P*ase1*-GFP-Fis1:KanR Ost4-tdTomato:NatR *Pnmt41*-Yta4(W165A/F166A)-13Myc:*ura+* *ade6*-m210 *leu1*-32 h? | This study |
| CF.13020 | *yta4*Δ:KanR *Pase1*-GFP-Fis1:KanR Ost4-tdTomato:NatR *Pnmt41*-Yta4(E192Q)-13Myc:*ura+* *ade6*-m210 *leu1*-32 h? | This study |
| **S3 Fig** |  |  |
| CF.9210 | *Pase1*-GFP-Fis1:KanR *ade6*-m210 *leu1*-32 *ura*-294- h- | This study |
| CF.9380 | *yta4*Δ:KanR *Pase1*-GFP-Fis1:KanR *ade6*-m210 *leu1*-32 *ura4*-294- h+ | This study |
| CF.9849 | *yta4*Δ:KanR *Pase1*-GFP-Fis1:KanR P*yta4*-Yta4(WT)-13Myc:*ura+* *ade6*-m210 *leu1*-32 h+ | This study |
| CF.9850 | *yta4*Δ:KanR *Pase1*-GFP-Fis1:KanR *Pyta4*-Yta4(W165A/F166A)-13Myc:*ura+* *ade6*-m210 *leu1*-32 h+ | This study |
| CF.9851 | *yta4*Δ:KanR *Pase1*-GFP-Fis1:KanR *Pyta4*-Yta4(E192Q)-13Myc:*ura+* *ade6*-m210 *leu1*-32 h? | This study |
| CF.8648 | *Pase1*-Dnm1-GFP:*leu+* *ade6*-m210 *ura4*-294-h? | This study |
| CF.9218 | *yta4*Δ:KanR *Pase1*-Dnm1-GFP:*leu+* *ade6*-m210 *ura4*-294-h? | This study |
| CF.13214 | *yta4*Δ:KanR *Pase1*-Dnm1-GFP:*leu+* *Pyta4*-Yta4(WT)-13Myc:*ura+* *ade6*-m210 h? | This study |
| CF.12989 | *yta4*Δ:KanR *Pase1*-Dnm1-GFP:*leu+* *Pyta4*-Yta4(W165A/F166A)-13Myc:*ura+* *ade6*-m210 h? | This study |
| CF.12990 | *yta4*Δ:KanR *Pase1*-Dnm1-GFP:*leu+* *Pyta4*-Yta4(E192Q)-13Myc:*ura+* *ade6*-m210 h? | This study |
