# Supplementary material for: The AAA-ATPase Yta4/ATAD1 interacts with the mitochondrial divisome to inhibit mitochondrial fission
Source: PLoS Biol. 2023 Aug 17;21(8):e3002247. doi: 10.1371/journal.pbio.3002247 (PMC10465003; doi:10.1371/journal.pbio.3002247)
Supplement: Attachment — Submitted filename: 01response to reviewers-Mar5 2023.docx [file pbio.3002247.s020.docx]

**Responses to the editorial and reviewers’ comments**

We are grateful to the editor and the two reviewers for their enthusiasm about our work and would like to thank them for providing the insightful suggestions. We have taken the comments seriously and made a strong effort to carefully address all concerns by performing a great deal of biochemical and cell biological work. Specifically, we have revised 12 figures (Figs. 1B, 1D, 1G, 3E, 8B, 8C, 10A, 10B, 10C, 11A, 11B and Fig 12) and added 20 new figures (Figs. 5A, 5B, 5C, 5D, 6C, 7A, 7B, 7C, 7D, 8A, 8D, 8E, 8F, 9A, 9B, 9C, 9D, 11C, 11D, and 11E). In addition, we have added 18 new supplemental figures (Figs S1A, S1B, S1C, S1D, S2, S3A, S3B, S3C, S3D, S4A, S4B, S4C, S4D, S4E, S5A, S5B, S6A, and S6B).

A version of the tracked-change manuscript was uploaded, and for the ease of reading, the responses below are highlighted in blue. We hope that the revised data would satisfactorily address the concerns raised.

**Editorial comments:**

Thank you for your patience while your manuscript entitled "The AAA-ATPase Yta4/ATAD1 interacts with Dnm1 and Fis1 to inhibit mitochondrial fission" was peer-reviewed at PLOS Biology. Please also accept my sincere apologies for the delay in providing you with our decision. The manuscript has now been evaluated by the PLOS Biology editors, an Academic Editor with relevant expertise, and by two independent reviewers.

The reviews are attached below. As you will see, the reviewers are generally positive and find the conclusions interesting and novel, however they also think that the results are a bit preliminary and raise several concerns, suggesting a substantial number of experiments that are needed to strengthen the results. Reviewer 1 thinks that the mechanism underlying the regulation of mitochondria fission by Yta4 is confusing and that the final model needs to be fleshed out. Reviewer 2 would like you to analyse the function of Yta4 and its mutants at normal expression levels, rather than by overexpression, and is not convinced about the effects of Yta4 on Dnm1, suggesting several experiments to test if they are functional, among other experiments.

We would like to thank the reviewers and the editorial team for providing the helpful suggestions. As you would see in the revised manuscript, we have performed a significant amount of work to carefully address all concerns.

Specifically, 1) a series of rigorous biochemical assays were performed to clarify the proposed model (see new Figs 8-12 and S4-S6), 2) the function of Yta4 and its mutant were analyzed at the endogenous level (see S3 Fig), and 3) additional biochemical assays were performed to further test the effect of Yta4 on Dnm1 (see Figs 9A, 10, 11, S4, and S5A). We hope these additional data will satisfactorily address all concerns raised by the reviewers.

Based on the specific comments of the reviewers and following discussion with the Academic Editor, it is clear that a substantial amount of work would be required to meet the criteria for publication in PLOS Biology.

However, given our and the reviewers interest in your study, we would be open to inviting a comprehensive revision of the study that thoroughly addresses all the reviewers' comments and, in general, to strengthen the mechanism by which Yta4 acts on Dnm1 and Fis1 to inhibit mitochondrial fission. Considering the extent of revision that would be needed, we cannot make a decision about publication until we have seen the revised manuscript and your response to the reviewers' comments. Your revised manuscript would need to be seen by the reviewers again, but please note that we would not engage them unless their main concerns have been addressed.

We would like to thank the editorial team for the patience and for giving us sufficient time to perform the substantial amount of work.

**Reviewer 1:**

In this study, the authors used fission yeast as a model organism to study the role of a conserved mitochondrial outer membrane protein Yta4 (Msp1 in budding yeast and ATAD1 in mammals) in regulating mitochondrial dynamics.

Although previous observations by another group showed that Msp1 deletion alone in budding yeast does not cause significant mitochondrial morphology change, the authors showed that deletion of Yta4 in fission yeast causes significant mitochondrial fragmentation, similar to the effect caused by the deletion of ATAD1 in mammals (shown by another group). The authors then showed that the fragmentation takes place in a Dnm1-dependent manner. In the second half of this manuscript, the authors used a combination of imaging and immunoprecipitation assays to establish the functional interactions between Yta4, Dnm1 and Fis1 and reached a model where Yta4 interactions with both Dnm1 and Fis1 and such interactions inhibit excessive mitochondrial fission.

The authors made several key observations/conclusions in this paper. The observation that Yta4 regulates mitochondria dynamics by inhibiting excessive fission is exciting and well supported by experimental data. It points out that Yta4 as an evolutionarily conserved protein, has a role that was previously not well understood, and this role could be conserved in mammals, given that ATAD1 deletion also causes mitochondrial fragmentation.

While this observation is exciting, the mechanism by which Yta4 regulates mitochondria fission is still confusing, especially the functional relationship between three key components: Yta4, Dnm1 and Fis1. The manuscript needs more experiments to strengthen the mechanism part. Also, the final model needs to be more clearly spelled out. The current model figure is not informative and needs a lot of improvement. Below are my detailed comments:

We would like to thank the reviewer for the positive comments. To further test our proposed model, we have followed the suggestions to perform rigorous biochemical assays (Figs.8-11 and S4-6). In addition, we have included Mdv1, the bridging protein between Fis1 and Dnm1 (Bui et al., 2012; Griffin et al., 2005; Karren et al., 2005; Koirala et al., 2010; Lackner et al., 2009; Zhang and Chan, 2007), in the experiments. For details, see the responses below. Based on the new data and the reviewer’s suggestion, the model was clarified and drawn (Fig. 12).

Major comments:

My main comments are on Figures 4, 5 and 6, where the authors tried to establish the functional relationships between Yta4, Dnm1 and Fis1.

(1) In Figure 4, the authors showed that overexpression of either a catalytic-dead mutant (Yta4(EQ)) or a translocation-defective mutant (Yta(AA)) of Yta4 causes dislocation of Dnm1 from the mitochondria without dislocalizing Fis1. Given that Dnm1 is recruited to the mitochondria by Fis1, this result suggests that Yta4 could destabilize the interaction between Dnm1 and Fis1, and this destabilization is not dependent on the enzymatic activity or the translocase activity of Yta4. The authors later speculated in the discussion that this implies that Yta4 could dislocate Dnm1 through a direct interaction with Dnm1, which is confusing, because if Yta4(EQ) is able to interact with Dnm1, it should be able to help Dnm1 localize to the mitochondria. So, taking the results of Figure 4, perhaps an explanation to this result that would make more sense is that Yta4's effects of the Dnm1-Fis1 complex is twofold: first, it removes Fis1 from the mitochondria, keeping the homeostatic balance of Fis1 on the outer membrane, and this function is dependent on the translocase activity. Second, its interaction with Fis1 inhibits Dnm1 from interacting with Fis1, and this interaction does not require a translocase or ATPase activity, potentially because monomeric Yta4 could interact with Fis1. So, when the wild-type Yta4 is overexpressed, both Yta4's functions take place, resulting in the dislocation of both Fis1 and Dnm1 from the mitochondria. However, when a mutant version of Yta4 is expressed, it could no longer dislocase Fis1, but still able to inhibit Dnm1-Fis1 interaction, causing the observed dislocation of Dnm1, but not Fis1.

We thank the reviewer for pointing out the issue and for suggesting the model. As suggested by the reviewer, Mdv1, the bridging protein between Fis1 and Dnm1(Bui et al., 2012; Griffin et al., 2005; Karren et al., 2005; Koirala et al., 2010; Lackner et al., 2009; Zhang and Chan, 2007), should have been included in the original work. In fact, the missing of the Mdv1 data in the original work caused an incomplete understanding of the interplay between Yta4 and the mitochondrial divisome composed of Fis1, Mdv1, and Dnm1.

To test the model in a comprehensive manner, we involved Mdv1in the revised work. The reviewer’s model is supported by our new data that the interaction between Mdv1 and Dnm1 is impaired by the presence of Yta4 (Fig 9A) and that overexpression of Yta4(WT), Yta4(AA), or Yta4(EQ) has a similar effect on the localization of Fis1 and Mdv1(Fig 4E and 7A), but not Dnm1 (Fig 4D). Moreover, the interaction between Fis1 and Mdv1 appeared to be strong as the two proteins can be co-purified (Fig. 8F), and the presence of Yta4 did not appear to affect the association of Fis1 with Mdv1 (Figs 9C and S6). Hence, similar to the model proposed by the reviewer, the interaction between Yta4 and Mdv1 blocks the interaction of Mdv1 with dnm1 (Fig. 12, dashed square 2). In addition, the interaction between Mdv1 and Yta4 is independent of the translocase or ATPase activities of Yta4 (Fig. 8D, 8E, and 9A). The overexpression data (Fig. 4D, 4E, and 7A) can be explained by the revised model, shown in Fig 12.

We would like to thank the reviewer again for raising this important point.

(2) In Figure 5, both in vivo and in vitro pull-downs were performed to examine the physical interaction between Yta4 and Dnm1/Fis1. As the nucleotide-bound state is key to a AAA protein's oligomeric state and function, the authors should do these experiments with nonhydrolyzable nucleotides added and compare to the conditions without nucleotides, as this will help examine whether hexamer formation will enhance these interactions, especially for the one between Yta4 and Fis1. Since the authors' model is that Yta4 helps to destabilize the Fis1-Dnm1 complex, an in vitro assay using the same setup described in this figure should be done to test whether that is the case (pre-immobilize Fis1 to beads and bind Dnm1 to it, and then add Yta4 and ATP to watch dissociation of the Dnm1), and whether that is dependent on the presence of ATP.

This is another very important point raised by the reviewer. In the revised manuscript, we have followed the suggestions to perform rigorous biochemical experiments.

In fact, Yta4 contains a transmembrane domain at its N-terminus (see the diagram in Fig. 8B). Therefore, it is challenging to produce the full-length recombinant protein of Yta4, and we failed to express and purify full-length Yta4 in vitro. Instead, a truncated version of Yta4 lacking the transmembrane domain (i.e., Yta4(∆TM)), which exists mainly as monomers (S4A, S4B, and S4D Fig) and does not have ATPase activity (S4E Fig), can be purified in vitro. It has been shown previously that a hexameric version of Msp1, the budding yeast counterpart of Yta4, can be generated by fusing a truncated version of Msp1 lacking the transmembrane domain (Msp1(∆TM)) to the hexamerization scaffold protein Hcp1 (i.e., Hcp1-Msp1(∆TM)) (Castanzo et al., 2020). Hcp1-Msp1(∆TM) exhibits ATPase, translocase, and robust unfoldase activities (Castanzo et al., 2020). We followed a similar procedure to produce Hcp1-Yta4(∆TM), which is a hexamer and shows an ATPase activity (S4C, S4D, and S4E Fig). Using the monomeric and hexameric versions of Yta4, i.e., Yta4(∆TM) and Hcp1-Yta4(∆TM), the biochemical assays, proposed by the reviewer, were performed.

First, we systematically tested the interactions between Yta4, Fis1, Mdv1, and Dnm1 by GST pull-down assays (three independent experiments with the controls GST alone and glutathione resins only as baits) (See Fig. 8). We confirmed that the C-terminal WD domain of Mdv1 interacts with Dnm1 (Fig. 8E) (Koirala et al., 2010; Naylor et al., 2006; Tieu et al., 2002) while the N-terminus of Mdv1 interacts with Fis1 (Fig. 8F) (Karren et al., 2005; Koirala et al., 2010; Zhang and Chan, 2007). We further used Yta4(∆TM) to test its interaction with Fis1(∆TM) (a truncated mutant lacking the transmembrane domain), Mdv1(WD) (the C-terminal WD domain of Mdv1), and Dnm1. All tests, but not the controls, exhibited positive interactions (Fig 8B, 8C, and 8D). These results suggest that Yta4 physically interacts with Dnm1, Mdv1, and Fis1 in an ATPase and hexamerization-independent manner in vitro. The result also reflects the low specificity of recognition of client proteins by ATAD1/Msp1/Yta4, which is consistent with the findings that Msp1/ATAD1 interacts with different types of proteins, including tail-anchored (TA) proteins (Chen et al., 2014) (Dederer et al., 2019) (Li et al., 2019) and non-TA proteins (Zhang et al., 2011) (Weidberg and Amon, 2018) (Aweida and Cohen, 2022).

Second, we tested whether hexamerization affects the interaction of Yta4 with Dnm1 and Fis1, using the monomeric and hexameric versions of Yta4, i.e., Yta4(∆TM) and Hcp1-Yta4(∆TM). Given that Yta4 interacts with Dnm1 in an ATPase-independent manner (Figs 8B and S4E), we tested the interactions of Yta4(∆TM) and Hcp1-Yta4(∆TM) with Dnm1 in the absence of ATP (S5A Fig). The result showed that hexamerization did not appear to significantly affect the interaction between Yta4 and Dnm1. Since Fis1 is a tail-anchored protein and overexpression of Yta4 dislocated Fis1 in an ATPase-dependent manner (Figs 4E and S1D), we tested the interactions of Yta4(∆TM) and Hcp1-Yta4(∆TM) with Fis1(∆TM) in the presence of ATP (S5A Fig). Interestingly, the result showed that hexamerization of Yta4 enhanced the interaction of Yta4 with Fis1(∆TM) and that the presence of ATP weakened the interaction between Yta4 and Fis1. Given that ATP hydrolysis promotes the motor activity of Msp1/Yta4 (Wang and Walter, 2020), we speculated that the dynamic nature of the Yta4 motor domain may be a main cause of the weakened interaction between Yta4 and Fis1 in vitro in the presence of ATP and in the absence of membranes.

Finally, we tested how the presence of Yta4 affects the interactions of Mdv1 with Dnm1 and Fis1 by GST pull-down assays, as suggested by the reviewer. As shown in Fig. 9A, the presence of Yta4(∆TM) significantly impaired the interaction between Mdv1 and Dnm1 but did not appear to affect the interaction between Mdv1 and Fis1 (S6A Fig). In addition, the presence of Hcp1-Yta4(∆TM) did not affect the interaction between Mdv1 and Fis1 (Fig 9C). These results support the model, similar to the one suggested by the reviewer, that Yta4 interacts with Mdv1 to block the recruitment of Dnm1 (Fig 12, dashed square 2).

(3) In Figure 6B, the authors examined the effect of Yta4 binding to the GTPase activity of Dnm1. Could the authors specify if Yta4 binding changes the Vmax of the reaction? If not, Yta4 binds in a competitive mode, without changing the inherent enzymatic capacity of Dnm1. It would be helpful to distinguish mechanistically (with the data the authors already have) whether Yta4 is a competitive or noncompetitive inhibitor.

Dnm1 forms heterogeneous oligomers in vitro (Ingerman et al., 2005; Lackner et al., 2009) and the concentration of Dnm1 appeared to play a crucial role in dictating the states of Dnm1 oligomerization. Therefore, to precisely assess the effect of Yta4 on the GTPase activity of Dnm1, we carefully performed parallel colorimetric assays again, using a single batch of recombinant Dnm1 and His-Yta4(∆TM) (Fig 10B and 10C).

First, we performed a control experiment (three independent repeats) to test the GTPase activity of Dnm1 and His-Yta4(∆TM) and the buffer used to keep His-Yta4(∆TM) and Dnm1. The result showed that only Dnm1 showed a significant GTP activity (Fig 10B).

We then carefully tested how His-Yta4(∆TM), at different concentrations, affects the GTPase activity of Dnm1 (three independent experiments) (Fig 10C). In the presence of 5 µM and 10 µM His-Yta4(∆TM), the K_m_ value of Dnm1 was increased ~3 and ~6-folds, indicating that His-Yta4(∆TM) reduced the affinity of Dnm1 for GTP. In contrast, the V_max_ value was not significantly affected by His-Yta4(∆TM), suggesting that His-Yta4(∆TM) did not affect the rate of Dnm1-mediated GTP hydrolysis (Fig 10B). Collectively, these results suggest that Yta4 is a competitive inhibitor of Dnm1.

(4) Figure 6C suggests that Yta4 can compete out GDP or GTP from binding to Dnm1, but it fails to compete out GMPPNP. There is no explanation to this result. Is that because GMPPNP is known to bind Dnm1 more strongly? It's confusing what the GMPPNP group shows here. Also, it would help to have a group without any nucleotide, as a negative control.

To test the effect of Yta4(∆TM) on Dnm1 self-assembly, we followed the experimental procedure, described previously (Lackner et al., 2009), to perform velocity sedimentation in our original work (original Fig 6C). Consistent with the data shown in (Lackner et al., 2009) (see Fig 3A in the paper), in the absence of Yta4, Dnm1 was present in the pellets of the GDP, GTP, and GMP-PCP-treated samples with the GMP-PCP-treated sample showing the highest amount of Dnm1 in the pellet. Note that GMP-PCP is a nonhydrolyzable GTP analog, which has been shown to promote the formation of Dnm1 rings and spiral-like structures (Ingerman et al., 2005). As suggested by the reviewer, we have included a group without any nucleotide (see Figure R1 below). The result was similar. These confirmed that Dnm1 spontaneously self-assembled in vitro regardless the presence of nucleotides.

In the original Fig 6C and 6D and Fig R1 below, the presence of His-Yta4(∆TM) significantly reduced the amount of Dnm1 in all pellets except in the pellet of the GMP-PCP-treated sample.

1) The results suggest that His-Yta4(∆TM) affects Dnm1 self-assembly, likely preventing the formation of high-order structures (i.e., filament-like structures) in the samples treated with GDP/GTP or without nucleotides. In fact, our data collected by negative staining electron microscopy support the conclusion. As shown in new Fig 11C, Dnm1 forms small filament -like structures in the absence of GMP-PCP and such structures were hardly seen when His-Yta4(∆TM) was added.

2) We employed negative staining electron microscopy to examine Dnm1 samples treated with 200 µM GMP-PCP. As shown in Figure R2 below, the presence of 200 µM GMP-PCP promoted the formation of large spiral-like structures. Note that in the velocity sedimentation assays, proteins were mixed and incubated before nucleotides were added (see the procedure in new Fig 11A, below the graph). We felt that excessive GMP-PCP (e.g., at 200 µM) could outcompete His-Yta4(∆TM) (at 5 µM) to promote the formation of Dnm1 (at 0.5 µM) spirals.

**Figure R1. Testing Dnm1 self-assembly by velocity sedimentation assays.** (A) SDS–PAGE was performed to analyze the effect of His-Yta4(∆TM) on Dnm1 assembly in the absence of nucleotides. A group of samples lacking His-Yta4(∆TM) were included. S and P represent the supernatant and pellet of the indicated samples after centrifugation, respectively. Note that the presence of His-Yta4(∆TM) significantly reduced the amount of Dnm1 in the pellet fractions. Three independent experiments were performed. (B) Quantification of the percentage of Dnm1 in the pellet. The top of the column indicates the mean while error bars represent S.D. Student’s t test was used to calculate *p* values.

Reviewer 2 was concerned about the data shown in the original Fig 6C, stating “However, it is not clear if Dnm1 in the pellet reflects filament formation or just aggregate formation.”. Note that Dnm1 mediates mitochondrial fission likely by forming rings/spirals on a mitochondrion (Ingerman et al., 2005). Therefore, to avoid confusion, we did not include the samples treated with GDP/GTP in the revised manuscript. Instead, based on the assumption that Dnm1 forms high-order structures in a GMP-PCP concentration-dependent manner, we titrated the concentration of GMP-PCP and examined the effect of His-Yta4(∆TM) on Dnm1 assembly promoted by GMP-PCP at different concentrations (i.e., 0, 5, 50, and 100 µM). As shown in new Fig 11A and 11B, the increase in GMP-PCP concentration increased the amount of Dnm1 in the pellets, and the presence of His-Yta4(∆TM) significantly reduced the amount of Dnm1 in the pellets. These results unambiguously suggest that His-Yta4(∆TM) impaired Dnm1 assembly by competing with GMP-PCP, which is also supported by the GTPase kinetics assays shown in Fig 10. To further test this conclusion, we employed negative staining electron microscopy to examine the samples treated with 100 µM GMP-PCP. As shown in Fig 11C-11E, the presence of 100 µM GMP-PCP mainly promoted the formation of Dnm1 rings but some small Dnm1 spirals were also detected under this condition, and His-Yta4(∆TM) was quite efficient in preventing the formation of Dnm1 rings and spirals. Hence, our data unambiguously suggest Yta4 can inhibit Dnm1 assembly.

**Figure R2. Examining Dnm1 by negative staining electron microscopy.** Representative images of negative staining electron microscopy. Dnm1 alone was incubated in a buffer containing or lacking 200 µM GMP-PCP. Images were acquired at a magnification of 62,000. Note that Dnm1 filament-like/aggregates structure was found in the sample without GMP-PCP while large spiral-like structures were detected in the samples containing 200 µM GMP-PCP. Scale bar, 100 nm.

(5) The model figure (Figure 7) needs a lot of improvement. Currently, it is extremely confusing what it is trying to convey. A good way to make this figure could be describing the normal versus the Yta4 deletion scenarios separately, and spell out clearly which component interacts with which component and which interaction helps dislocate which component etc.

We agreed with the reviewer that the original model is confusing. Therefore, based on our new data and the reviewer’s suggestions, we have improved the model (see new Fig 12). Specifically, 1) the normal and Yta4-deletion scenarios were shown separately, and 2) we clearly illustrated in the diagram the effects of Yta4 on the three components of the mitochondrial divisome.

(6) The authors mentioned that Dnm1 is recruited to the mitochondria by Fis1, through Cav4/Mdv1. However, there is no examination of Yta4's effect on Cav4/Mdv1's mitochondrial localization throughout this study. To strengthen the mechanistic part of this study, the authors should examine the effect of Yta4 deletion and overexpression on Cav4/Mdv1, and possibly test their physical interactions using the already established in vitro assay, to further dissect the role of Yta4 in the stabilization/destabilization of this big complex and how that leads to the observed morphological change in the organelle.

We agreed with the reviewer that missing Mdv1 was a main drawback of our original work. Hence, we have followed the reviewer’s suggestions to involve Mdv1 in our revised work. Specifically, 1) testing the interaction between Yta4(∆TM) and Mdv1(WD) (Fig 8D), 2) confirming the interaction between Dnm1 and Mdv1(WD) (Fig 8E), 3) confirming the interaction between Fis1(∆TM) and Mdv1(NT) (Fig 8F), 4) testing the competitive effect of Yta4(∆TM) on the interaction between Dnm1 and Mdv1(WD) (Fig 9A), 5) testing the effect of Hcp1-Yta4(∆TM) on the interaction between Fis1(∆TM) and Mdv1(NT) (Fig 9C and S6). Moreover, the effect of overexpression of Yta4(WT)/ Yta4(AA)/ Yta4(EQ) on the localization of Mdv1to mitochondria was tested (Fig 7A), and the in vivo interaction of Yta4 with Mdv1 was tested by co-immunoprecipitation (Fig 7D). The data obtained unambiguously support the new model proposed in Fig 12.

We agree with the reviewer that the original model is confusing. Therefore, based on our new data and the reviewer’s suggestions, we have improved the model in the revised manuscript (see new Fig 12). Specifically, 1) the normal and Yta4-deletion scenarios were shown separately, and 2) we clearly illustrated in the diagram the effects of Yta4 on the three components of the mitochondrial divisome.

Minor comments:

(1) Msp1 is misspelled as Mps1 three times. Twice in page 3 and once in page 6.

Corrected

(2) The bottom panel of Figure 3A showed mitochondrial constriction (but not fission) in the absence of Dnm1. Can the authors comment on the cause of that?

Yes, this is a very interesting phenotype. Some *dnm1*∆ cells displayed the phenotype of mitochondrial constriction after the treatment with 2 µM FCCP, and the phenotype became more apparent for *yta4*∆*dnm1*∆ cells treated with 2 µM FCCP (Figure 3A, the bottom right images).

The ER mediates mitochondrial constriction at the contact site, and during and/or after mitochondrial constriction, Dnm1/Drp1 is recruited to the contact site to promote the ultimate mitochondrial fission (Friedman et al., 2011). In yeasts, the ERMES complex, composed of Mdm10, Mdm34, Mmm1, and Mdm12, is involved in the formation of the ER-mitochondria contact site. Mdm10 is inserted into the mitochondrial outer membrane while Mdm34 is peripheral to the mitochondrial outer membrane and Mdm12 is cytosolic (Lang et al., 2015). We (Rasul et al., 2021) and others (Kornmann et al., 2011; Yamano et al., 2010) identified ERMES regulatory proteins and found that these regulatory proteins (e.g., Emr1, Gem1, Tom7) possess a single transmembrane domain and are inserted into the mitochondrial outer membrane. Given the canonical role of Yta4/Msp1 in dislocating tail-anchored proteins from the mitochondrial outer membrane, we speculated that Yta4 may also regulate the contact between the ER and mitochondria through maintaining the homeostatic balance of the ERMES components and ERMES regulatory proteins on the mitochondrial outer membrane. The depolarization of mitochondria by FCCP treatment promotes mitochondrial fission (Figure 3A). We speculate that the contact between ER and mitochondria is involved in the FCCP-induced mitochondrial fission, and the absence of Yta4 may enhance the function of the ERMES complex and consequently leads to an enhancement of ER wrapping on mitochondria. In this scenario, the absence of the mitochondrial fission factor Dnm1 allows for detection of mitochondrial constriction. At the moment, we are dissecting the role of Yta4 in regulating the ERMES complex and hope to publish the work in a follow-up study.

The statement below has been added in the result section in the revised manuscript.

“We noticed that mitochondria underwent constriction, but not fission, in *yta4*Δ*dnm1*Δ cells after the treatment with 2 µM FCCP (Fig 3A, bottom panel). Since the wrapping of ER on mitochondria mediates mitochondrial constriction and promotes the Dnm1-dependent mitochondrial fission [28], we speculated that Yta4 may also regulate the contact between the ER and mitochondria.”

(3) Figure 1B: I assume the top-right panel refers to mitochondria per cell, whereas the bottom panels refer to junctions per mitochondria (not per cell)? If so, the authors should specify this in the text.

The indicated numbers are mitochondrial number per cell, branch number per cell, and junction number per cell. This has been clarified in the figure legend.

(4) Figure 1G: should the bottom panel be "fusion" instead of "fission"?

Yes, it is “fusion”. It has been corrected.

(5) There are a few places where the authors observed clumped mitochondria. For example, in Figure 4D, expression of Yta4 (EQ) leads to clumping. Is that a sign of inhibited fusion? The authors should comment on the cause of clumping, and why that is observed only when the dead mutant is expressed by not in the Yta4 deletion cells.

The phenotype of the clumped mitochondria is interesting and was seen only when Yta4(EQ) or Yta4(AA) mutant was overexpressed.

The phenotype could be due to the inhibition of mitochondrial fusion but is unlikely due to only the inhibition of mitochondrial fusion because we have shown previously that multiple (>=2) small mitochondrial aggregates form in cells lacking the mitochondrial fusion factor Fzo1 (Dong et al., 2022). Generally, one mitochondrial clump formed in cells overexpressing Yta4(EQ) or Yta4(AA) (Figs 4D, 4E, 7A, S1C, and S1D). Since Yta4(EQ) is ATP hydrolysis-deficient and Yta4(AA) is translocation-deficient, the association of Yta4(EQ) or Yta4(AA), but not wild-type Yta4, with the tail-anchored client proteins, which are inserted into the mitochondrial outer membrane, may remain stable. The stable association may then promote fusion/clustering of neighboring mitochondrial outer membranes. Ultimately, mitochondrial clumps result.

The speculative view has been added in the result section as follows:

“We speculated that Yta4(EQ) and Yta4(AA) may stably associate with their neighboring tail-anchored client proteins that reside on the mitochondrial outer membrane, and consequently, the stable association may promote fusion/clustering of neighboring mitochondria and lead to mitochondrial aggregation.”

Since Yta4 functions to inhibit the mitochondrial divisome, Yta4-deletion should promote mitochondrial fission, i.e., mitochondria become fragmented (See Fig 1).

(6) Figure 5D and 5F: in the GST blot, what is the top band in the third lane? It is only present in the control lane but not in the experiment lane.

The top band in the lane of the GST control (new Fig 8B-8E) should be a nonspecific band recognized by our antibody against GST because similar bands were detected in the GST control lane in all GST pull-down experiments (Figs 8D-8E, 9, S5B, and S6A). We have clearly marked the nonspecific band with red asterisks and explained in the revised figure legends.

**Reviewer 2**

Msp1/ATAD1 in budding yeast and mammals as an AAA-ATPase in the mitochondrial outer membrane (OM) mediates the quality control of mistargeted tail-anchor (TA) protein on the mitochondrial surface; Msp1 extracts mistargeted TA proteins from the mitochondrial OM and transfers them to the ER, where mistargeted proteins are subjected to the ER protein quality control. In the present study, He et al. report a new possible role of Yta4, an Msp1 homolog in fission yeast, in the control of the mitochondrial morphology. The observation is interesting and may attract the interest of the broad readership of the journal. However, the manuscript has many points to be improved.

We would like to thank the reviewer for the support and for providing the helpful suggestions below.

Major points.

1) It is of interest if the observed effects of Yta4 on Dnm1 functions reflect the possible ability of Yta4 to extract Fis1 (and Dnm1?) from the mitochondrial OM. That is, does Yta4 indeed extract a fraction of normal, not mistargeted Fis1, from the OM to suppress the level of recruited Dnm1? This can be tested by following the fate of Fis1 upon expression of Yta4 in yta1Δ cells.

We have drawn a diagram to illustrate the mitochondrial divisome composed of Fis1, Mdv1, and Dnm1 in new Fig 12. During the revision, a large amount of biochemical work (See new Figs 8-11 and S4-S6) was carried out to collect evidence to support the model. As shown in Fig 8A, Fis1 is a typical tail-anchored protein carrying a C-terminal transmembrane domain while both Dnm1 and Mdv1 do not have any transmembrane domain. Fis1 recruits Dnm1 to the mitochondrial outer membrane via the bridging protein Mdv1 (Bui et al., 2012; Griffin et al., 2005; Karren et al., 2005; Koirala et al., 2010; Lackner et al., 2009; Zhang and Chan, 2007). We have followed reviewer 1’s suggestion to involve Mdv1 in the present study. Overall, the model is that Yta4 inhibits the function/assembly of the mitochondrial divisome by multiple mechanisms, shown in Fig 12.

To address the question raised here, we followed the reviewer’s suggestion to follow the fate of Fis1 and Dnm1 upon expression of Yta4 in *yta4*∆ cells (See new S1 Fig). Specifically, Yta4(WT)/Yta4(AA)/Yta4(EQ)-13Myc was expressed in *yta4*∆ cells carrying either Dnm1-GFP or GFP-Fis1 from the *nmt41* promoter, which is suppressible by thiamine (see S1A Fig). The cells were first cultured in EMM5S medium containing 0.3 µM thiamine (to suppress the expression of Yta4-13myc), then collected and washed with thiamine-free EMM5S medium, and cultured in thiamine-free EMM5S medium for 3, 6, 9, 32 hours. At 3, 6, 9, 32 hours after the release, the cells were collected and analyzed by western blotting (S1B, Fig, to monitor the expression of Yta4-13myc) and the localization of GFP-Fis1 (S1D Fig) or Dnm1-GFP (S1C Fig).

The western blotting results showed that the levels of Yta4(WT)/Yta4(AA)/Yta4(EQ)-13Myc were detectable after 6 hours of the release of the suppression and increased over time (S1B Fig). Consistently, the expression of Yta4(WT), but not Yta4(AA) and Yta4(EQ), dislocated GFP-Fis1 from mitochondria (S1D Fig), which was consistent with the data shown in Fig 4E. We also noticed that the signals of GFP-Fis1 in the cytoplasm became much stronger when Yta4(WT) was expressed, supporting the model that Yta4 extracts Fis1 from mitochondria. Moreover, cell fractionation experiments showed that GFP-Fis1 in the Yta4(WT)-overexpressing cells was present in the cytosolic fraction (new Fig 5D). Similar results were found for Dnm1-GFP (S1C Fig). Fis1 forms a complex with Mdv1 (Karren et al., 2005; Zhang and Chan, 2007), and the interaction between Fis1 and Mdv1 appeared to be strong (Figs 8F, 9C, and S6). These results predict that overexpression of Yta4(WT)/Yta4(AA)/Yta4(EQ) should have a similar effect on the localization of Mdv1 and Fis1. Consistently, overexpression of Yta4(WT), but not Yta4(AA) and Yta4(EQ), dislocated GFP-Mdv1 from mitochondria (new Fig 7A). Overall, our data unambiguously support the model that Yta4 can extract Fis1 from mitochondria.

In relation to this, it is strange that, while the level of GFP-Fis1 increased with overexpressed WT Yta4 (Fig. 4C), the GFP-Fis1 signal decreased under the microscope (Fig. 4E). If Fis1 was extracted from the OM but not degraded, is the extracted Fis1 transferred to the ER?

As shown in Figs 5D and S1D, after extracted by Yta4, GFP-Fis1 localized in the cytoplasm whereas before extracted, GFP-Fis1 was present on mitochondria as foci (concentrated Fis1). GFP-Fis1 signals were homogenous in the cytoplasm and did not appear to overlap with ER signals (S2 Fig). The homogeneity of GFP-Fis1 signals in the cytoplasm may give the impression that GFP-Fis1 signals decreased in the Yta4(WT)-overexpressing cells under the microscope.

2) Since overexpression of Yta4 could cause, like in the case of Msp1, secondary effects through perturbed TA protein trafficking, functions of Yta4 and its mutants should be analyzed at their normal expression levels by using the YTA4 own promoter (Fig. 4). Co-IP experiments should be performed with cells with normal levels of Yta4 or WT cells, not the cells with overexpressed Yta4 in Fig. 5C.

We agree with the reviewer that secondary effects could be an issue. However, overexpression studies are likely the ideal approach in this case. The reasons are as follows:

As shown in the present study, Yta4 functions to inhibits the mitochondrial divisome (Fig 12). In this scenario, the absence of Yta4 releases the inhibitory effect of the mitochondrial divisome, promoting excessive mitochondrial fission and ultimately mitochondrial fragmentation. This phenotype was confirmed by our microscopic data (Fig 1). Therefore, expression of Yta4(WT) at the endogenous level should rescue the mitochondrial phenotype caused by the absence of Yta4, which is true (Figs 1C, 1F, S3A and S3C).

Our biochemical data showed that the monomeric form of Yta4 (see S4 Fig) impaired the interaction between Mdv1 and Dnm1 in a competitive manner (Fig 9A) and inhibited the GTPase activity of Dnm1 in a competitive manner (Fig 10) but did not appear to affect the interaction between Fis1 and Mdv1. These data suggest that the inhibitory effects of Yta4 on the interaction between Dnm1 and Mdv1 and Dnm1 GTPase activity do not depend on the enzymatic or translocase activity of Yta4. In other words, if Yta4(WT), Yta4(AA), and Yta4(EQ) were expressed at the endogenous level, they would essentially have a similar inhibitory effect on the interaction between Mdv1 and Dnm1 and Dnm1 GTPase activity, and thus have a similar inhibitory effect on Dnm1-mediated mitochondrial fission.

In wild-type cells, in which Yta4 is expressed at the endogenous level, Fis1 is present only on mitochondria (note that a transmembrane domain is present at the C-terminus of Fis1) (Figs 5D and S3A). Based on our overexpression data, Yta4 extracts Fis1 in a manner dependent of the ATPase enzymatic activity (Figs 4E and S1D). Therefore, expression of Yta4(AA) and Yta4(EQ) at the endogenous level should not affect the mitochondrial localization of Fis1 and mitochondrial fission (note that as stated above, expression of Yta4(WT)/Yta4(AA)/Yta4(EQ) has a similar inhibitory effect on Dnm1). Collectively, in the present case, it is challenging to dissect the function of Yta4 on the mitochondrial divisome by expression of Yta4(WT)/Yta4(AA)/Yta4(EQ) only at the endogenous level.

Despite the discussion above, we agree with the reviewer that the functions of Yta4 and its mutants should be analyzed at the endogenous level. Therefore, we expressed Yta4(WT)/Yta4(AA)/Yta4(EQ) from the yta4 promoter in *yta4*∆ cells expressing either GFP-Fis1 and Dnm1-GFP (S3 Fig). Microscopic data are consistent with the predictions, stated above, that the localization of GFP-Fis1 and Dnm1-GFP did not appear to be affected by the expression of Yta4(WT)/Yta4(AA)/Yta4(EQ) at the endogenous level. Moreover, quantification of mitochondrial fission confirmed that the expression of Yta4(WT), Yta4(AA), or Yta4(EQ) at the endogenous level rescued the phenotype of excessive mitochondrial fission caused by the absence of Yta4 (S3 Fig). Therefore, to be able to discern the differential roles of Yta4(WT)/Yta4(AA)/Yta4(EQ) in regulating the mitochondrial divisome, overexpression experiments should be considered.

Finally, we followed the reviewer’s suggestion to perform co-IP using cells expressing Yta4 from its own promoter (at the endogenous level) (see new Fig 6C). The new co-IP data and the original data are similar: Yta4 interacts with Fis1 and Dnm1 in vivo.

3) In Fig. 6, the authors tested the effects of Yta4 on the GTPase activity of Dnm1 by using recombinant Dnm1 and Yta4. However, these experiments are problematic, and the results on the effects of Yta1 on Dnm1 functions appear preliminary.

We have followed the suggestions, provided by both reviewers, to improve the experimental designs and performed rigorous biochemical assays during the revision to address the concerns. See details below.

- It is not clear if Dnm1 and Yta4 are functional; they only tested the GTPase activity of Dnm1. Does Yta4 show the expected ATPase activity? Does purified Yta4 exist as a monomer or oligomer?

Yta4(∆TM), which lacks the transmembrane domain, is a monomer (See S4B and S4D Fig) and did not show the ATPase activity (See S4E Fig). To generate a Yta4 hexamer, we followed the procedure that was reported recently to generate a hexamer of the budding counterpart of Yta4 (i.e. Msp1) (Castanzo et al., 2020). Specifically, Yta4(∆TM) was fused to a hexameric scaffold protein Hcp1 to produce a hexamer, i,e, Hcp1-Yta4(∆TM). Size exclusion chromatography (S4C Fig) and cross-linking assays (S4D Fig) confirmed that similar to Hcp1-Msp1∆TM), reported recently (Castanzo et al., 2020), Hcp1-Yta4(∆TM) is a hexamer. Moreover, Hcp1-Yta4(∆TM) showed a significant ATPase activity (S4E Fig). The function of Dnm1 was tested again by careful GTPase kinetics assays (Fig 10B) and GMP-PCP-induced oligomerization assays (Fig 11C). The results suggest that Dnm1 is functional.

- The effects of Yta4 on possible filament formation of Dnm1 were tested by centrifugation in Fig. 6C. However, it is not clear if Dnm1 in the pellet reflects filament formation or just aggregate formation.

We agree with the reviewer that using only velocity sedimentation assays was not enough to reveal the states of Dnm1 assembly.

It has been shown that Dnm1 spontaneously forms filament-like structures in vitro and GMP-PCP, the nonhydrolyzable GTP analog, induces the formation of high-order Dnm1 spiral-like structures (Ingerman et al., 2005). In the revised work, we employed negative stain electron microscopy to examine Dnm1 in the absence and presence of GMP-PCP (at 100 µM, lower than the concentration of GMP-PCP used originally). As shown in Fig 11C, Dnm1 form short filament-like structures in the absence of nucleotides and rings or small spirals in the presence of 100 µM GMP-PCP. The presence of Yta4(∆TM) significantly reduced the number of Dnm1 rings/spirals induced by 100 µM GMP-PCP (Fig 11C-11E). Consistently, as shown by the

velocity sedimentation assays (Fig 11A and 11B), the increase in GMP-PCP concentration increased the amount of Dnm1 in the pellets, and the presence of His-Yta4(∆TM) significantly reduced the amount of Dnm1 in the pellets. These results, together with the results of the GTPase kinetics assays shown in Fig 10, unambiguously suggest that Yta4(∆TM) impaired Dnm1 assembly by competing with GMP-PCP.

- It is unclear why they tested only single concentrations of the proteins: Dnm1 = 0.5 uM and Yta4 =5 uM.

The concentration of Dnm1 at 0.5 µM was used in GTPase kinetics assays in the previously study (Lackner et al., 2009). Therefore, we used 0.5 µM Dnm1 in the GTPase kinetics assays. This information has been added in the result section of the revised manuscript.

We agreed with the reviewer that Yta4 at different concentrations should be tested. Therefore, in the new GTPase kinetics experiments, we used 0, 1, 5, and 10 µM Yta4(∆TM). Consistently, the increase in the concentration of Yta4(∆TM) enhanced the inhibitory effect of Yta4(∆TM) on the GTPase activity of Dnm1 (Fig 10C).

Dnm1 forms heterogeneous oligomers in vitro (Ingerman et al., 2005; Lackner et al., 2009) and the concentration of Dnm1 appeared to play a crucial role in dictating the states of Dnm1 oligomerization. Therefore, to precisely assess the effect of Yta4 on the GTPase activity of Dnm1, we carefully performed parallel colorimetric assays, using Dnm1 and His-Yta4(∆TM) from a single batch of purification (Fig 10B and 10C).

- The nucleotide-bound state of Dnm1 should affect the localization of Dnm1 to mitochondria as well. This should be considered.

We appreciate the suggestion. The following statement has added in the discussion of the revised manuscript.

“Considering the fact that Mdv1 preferentially interacts with GTP-bound Dnm1 and promotes Dnm1 assembly (Lackner et al., 2009), we speculate that the interaction between Yta4 and Dnm1 is dynamic but the dynamic interaction is sufficient to reduce the amount of GTP-bound Dnm1, preventing the recruitment of Dnm1 by Mdv1 (Fig 12, dashed square 2).”

- GTP may be hydrolyzed to GDP under the condition of Fig. 6C.

Dnm1 mediated mitochondrial fission likely by forming rings/spirals on a mitochondrion (Ingerman et al., 2005), and GMP-PCP induced the formation of rings/spirals in vitro (Fig 11C) (Ingerman et al., 2005). Therefore, to avoid confusion, as pointed out by the reviewer, we did not include the samples treated with GDP/GTP in the revised manuscript. Instead, we tested the effects of Yta4 on the formation of Dnm1 rings/spirals induced by GMP-PCP at different concentrations (new Fig 11A and 11B)

4) Fig. 4D suggests that Fis1 interacting with the Yta4 mutants cannot recruit Dnm1 to mitochondria. This should be directly tested.

As pointed out by reviewer 1, missing Mdv1, the bridging protein between Fis1 and Dnm1, is a major drawback of the original work. Therefore, we have made a strong effort to test the role of Yta4 in regulating the mitochondrial divisome after involving Mdv1 (see new data Figs 7-11 and S4-S6). Collectively, the data support the model as proposed in Fig 12.

Briefly, Yta4 does not affect the interaction of Fis1 with Mdv1 (Figs 9C and S6) but impairs the interaction between Mdv1 and Dnm1 (Fig 9A). This may be the reason why the localization of Dnm1 on mitochondria was impaired when the Yta4 mutants were overexpressed.

Minor points

Page 3 - Fmp32 (Put6) localization needs a reference of

<https://www.nature.com/articles/s41467-020-18704-1>

The reference has been cited in the revised manuscript.

Ftr1 should read Frt1, and Yst6 should read Ysy6.

Corrected.

Fig. 4D and E - Why mitochondria become aggregated with overexpressed mutant Yta1?

We have added the speculative view below in the result section of the revised manuscript to explain the phenotype.

“We speculated that Yta4(EQ) and Yta4(AA) may stably associate with their neighboring tail-anchored client proteins that reside on the mitochondrial outer membrane, and consequently, the stable association may promote fusion/clustering of neighboring mitochondria and lead to mitochondrial aggregation”

Fig. 4B and text - Kcat should read kcat.

As suggested by reviewer 1, we showed V_max_ in the revised manuscript.

Fig. 4E - The signals from Mito Tracker are too strong and saturated.

We generally scaled the images, from parallel experiments, in a consistent manner. Some mutant cells were stained strongly, which might cause the strong signals. Based on the display of the MitoTracker signals of wild type cells, we have slightly reduced the display of other cells.

Fig. 5A - Yta4-tdTomato as a homolog of Msp1 is expected to localize not only to mitochondria but also peroxisomes. However, peroxisomal localization of Yta4 is not obvious here. Why?

To address this question, we examined the localization of Yta4-tdTomato and Pex13-GFP (marking peroxisomes). As shown in Figure R3 below, peroxisomes appeared to largely overlap with mitochondria (indicated by Yta4-tdTomato). This made it challenging to distinguish the peroxisomal localization of Yta4.

**Figure R3. The localization of Yta4-tdTomato and Pex13-GFP.** Maximum projection images of cells expressing Pex13-GFP (a protein localizing to peroxisomes) and Yta4-tdTomato. Magnified images of the dashed region are shown at the bottom panel. Scale bar, 10 µm.

Fig. 5C - The positions of Yta4-13myc and GFP-Fis1 are not the same among the three gels for each. If the rightmost panel is to show that GFP IP can pull down Dnm1, it needs control without GFP-Fis1 expression.

We have followed the reviewer’s suggestion to perform co-IP using Yta4 expressed from its own promoter and added the suggested control (see new data in Fig 6).

Fig. 5D and E - Input controls are missing.

See new data in Fig 8.

**References**

Aweida, D., and S. Cohen. 2022. The AAA-ATPase ATAD1 and its partners promote degradation of desmin intermediate filaments in muscle. *EMBO Rep*. 23:e55175.

Bui, H.T., M.A. Karren, D. Bhar, and J.M. Shaw. 2012. A novel motif in the yeast mitochondrial dynamin Dnm1 is essential for adaptor binding and membrane recruitment. *J Cell Biol*. 199:613-622.

Castanzo, D.T., B. LaFrance, and A. Martin. 2020. The AAA+ ATPase Msp1 is a processive protein translocase with robust unfoldase activity. *Proc Natl Acad Sci U S A*. 117:14970-14977.

Chen, Y.C., G.K. Umanah, N. Dephoure, S.A. Andrabi, S.P. Gygi, T.M. Dawson, V.L. Dawson, and J. Rutter. 2014. Msp1/ATAD1 maintains mitochondrial function by facilitating the degradation of mislocalized tail-anchored proteins. *EMBO J*. 33:1548-1564.

Dederer, V., A. Khmelinskii, A.G. Huhn, V. Okreglak, M. Knop, and M.K. Lemberg. 2019. Cooperation of mitochondrial and ER factors in quality control of tail-anchored proteins. *Elife*. 8.

Dong, F., M. Zhu, F. Zheng, and C. Fu. 2022. Mitochondrial fusion and fission are required for proper mitochondrial function and cell proliferation in fission yeast. *FEBS J*. 289:262-278.

Friedman, J.R., L.L. Lackner, M. West, J.R. DiBenedetto, J. Nunnari, and G.K. Voeltz. 2011. ER tubules mark sites of mitochondrial division. *Science*. 334:358-362.

Griffin, E.E., J. Graumann, and D.C. Chan. 2005. The WD40 protein Caf4p is a component of the mitochondrial fission machinery and recruits Dnm1p to mitochondria. *J Cell Biol*. 170:237-248.

Ingerman, E., E.M. Perkins, M. Marino, J.A. Mears, J.M. McCaffery, J.E. Hinshaw, and J. Nunnari. 2005. Dnm1 forms spirals that are structurally tailored to fit mitochondria. *J Cell Biol*. 170:1021-1027.

Karren, M.A., E.M. Coonrod, T.K. Anderson, and J.M. Shaw. 2005. The role of Fis1p-Mdv1p interactions in mitochondrial fission complex assembly. *J Cell Biol*. 171:291-301.

Koirala, S., H.T. Bui, H.L. Schubert, D.M. Eckert, C.P. Hill, M.S. Kay, and J.M. Shaw. 2010. Molecular architecture of a dynamin adaptor: implications for assembly of mitochondrial fission complexes. *J Cell Biol*. 191:1127-1139.

Kornmann, B., C. Osman, and P. Walter. 2011. The conserved GTPase Gem1 regulates endoplasmic reticulum-mitochondria connections. *Proc Natl Acad Sci U S A*. 108:14151-14156.

Lackner, L.L., J.S. Horner, and J. Nunnari. 2009. Mechanistic analysis of a dynamin effector. *Science*. 325:874-877.

Lang, A., A.T. John Peter, and B. Kornmann. 2015. ER-mitochondria contact sites in yeast: beyond the myths of ERMES. *Curr Opin Cell Biol*. 35:7-12.

Li, L., J. Zheng, X. Wu, and H. Jiang. 2019. Mitochondrial AAA-ATPase Msp1 detects mislocalized tail-anchored proteins through a dual-recognition mechanism. *EMBO Rep*. 20.

Naylor, K., E. Ingerman, V. Okreglak, M. Marino, J.E. Hinshaw, and J. Nunnari. 2006. Mdv1 interacts with assembled dnm1 to promote mitochondrial division. *J Biol Chem*. 281:2177-2183.

Rasul, F., F. Zheng, F. Dong, J. He, L. Liu, W. Liu, J.Y. Cheema, W. Wei, and C. Fu. 2021. Emr1 regulates the number of foci of the endoplasmic reticulum-mitochondria encounter structure complex. *Nat Commun*. 12:521.

Tieu, Q., V. Okreglak, K. Naylor, and J. Nunnari. 2002. The WD repeat protein, Mdv1p, functions as a molecular adaptor by interacting with Dnm1p and Fis1p during mitochondrial fission. *J Cell Biol*. 158:445-452.

Wang, L., and P. Walter. 2020. Msp1/ATAD1 in Protein Quality Control and Regulation of Synaptic Activities. *Annu Rev Cell Dev Biol*. 36:141-164.

Weidberg, H., and A. Amon. 2018. MitoCPR-A surveillance pathway that protects mitochondria in response to protein import stress. *Science*. 360.

Yamano, K., S. Tanaka-Yamano, and T. Endo. 2010. Tom7 regulates Mdm10-mediated assembly of the mitochondrial import channel protein Tom40. *J Biol Chem*. 285:41222-41231.

Zhang, J., Y. Wang, Z. Chi, M.J. Keuss, Y.M. Pai, H.C. Kang, J.H. Shin, A. Bugayenko, H. Wang, Y. Xiong, M.V. Pletnikov, M.P. Mattson, T.M. Dawson, and V.L. Dawson. 2011. The AAA+ ATPase Thorase regulates AMPA receptor-dependent synaptic plasticity and behavior. *Cell*. 145:284-299.

Zhang, Y., and D.C. Chan. 2007. Structural basis for recruitment of mitochondrial fission complexes by Fis1. *Proc Natl Acad Sci U S A*. 104:18526-18530.
