# Supplementary material for: The AAA-ATPase Yta4/ATAD1 interacts with the mitochondrial divisome to inhibit mitochondrial fission
Source: PLoS Biol. 2023 Aug 17;21(8):e3002247. doi: 10.1371/journal.pbio.3002247 (PMC10465003; doi:10.1371/journal.pbio.3002247)
Supplement: Attachment — Submitted filename: Reviewers_comments.docx [file pbio.3002247.s021.docx]

Reviewers’ comments

The authors made significant improvements on the previous manuscript. Now, the model of Yta4’s regulation on mitochondrial fission consists of three parts: (1) Yta4 extracts Fis1 from the OMM. (2) Yta4 inhibits the interaction between Mdv1 and Dnm1 (3) Yta4 is a competitive inhibitor of the GTPase activity of Dnm1.

Despite the experiments added, I still have concerns over some of these claims.

For the first claim, I am convinced that Fis1 is a Yta4 substrated when Yta4 is overexpressed. However, the authors showed that under normal Yta4 expression level, Fis1’s localization is on the mitochondria, and not significant changes in localization was observed when Yta4 is deleted. To claim that Yta4 removes Fis1 from the OMM under regular expression levels, the authors will need to quantify the level of Fis1 on the OMM in WT and KO conditions. Only when there is a change in Fis1’s level on the OMM, can the authors claim that Yta4 removes Fis1 to regulate mito fission.

For the second claim, the authors showed the Yta4 inhibits oligomer formation of Dnm1, since that is presented as a substantial piece of data in the paper, the authors should clarify whether the inhibition of Dnm1-Mdv1 interaction observed is due to the inhibition of oligomerization of Dnm1. That is, whether oligomerization of Dnm1 is required for the Dnm1-Mdv1 interaction.

For the third claim, I am convinced that Yta4 inhibits the GTPase activity. But again, how is this related to the inhibition of oligomerization? Since the authors presented a three-fold mechanism here, they should make sure that the three reasons are orthogonal to each other. The role of Dnm1 oligomerization in yta4’s inhibition to its activity needs to be fleshed out.

Overall, I recommend more experiments and text revision to flesh out the model before acceptance of publication.
