# Supplementary material for: The AAA-ATPase Yta4/ATAD1 interacts with the mitochondrial divisome to inhibit mitochondrial fission
Source: PLoS Biol. 2023 Aug 17;21(8):e3002247. doi: 10.1371/journal.pbio.3002247 (PMC10465003; doi:10.1371/journal.pbio.3002247)
Supplement: Attachment — Submitted filename: 01response to reviewers-2023-June2.docx [file pbio.3002247.s022.docx]

**Responses to the reviewers’ comments**

We greatly appreciate reviewer 1’s further suggestions. Following the suggestions, we have performed additional experiments and have revised the text accordingly. Specifically, new supplementary figures specified below were added to address the three concerns:

1) Point 1, new S3E Fig
2) Point 2, new S6A-S6C Fig
3) Point 3, new S8 Fig

For ease of reading, the responses below are highlighted in blue. We hope that the revised manuscript is acceptable for publication.

**Reviewers’ comments**

The authors made significant improvements on the previous manuscript. Now, the model of Yta4’s regulation on mitochondrial fission consists of three parts: (1) Yta4 extracts Fis1 from the OMM. (2) Yta4 inhibits the interaction between Mdv1 and Dnm1 (3) Yta4 is a competitive inhibitor of the GTPase activity of Dnm1.

We are grateful for the recognition of our efforts.

Despite the experiments added, I still have concerns over some of these claims.

We have performed additional experiments to address the three concerns listed below.

For the first claim, I am convinced that Fis1 is a Yta4 substrated when Yta4 is overexpressed. However, the authors showed that under normal Yta4 expression level, Fis1’s localization is on the mitochondria, and not significant changes in localization was observed when Yta4 is deleted. To claim that Yta4 removes Fis1 from the OMM under regular expression levels, the authors will need to quantify the level of Fis1 on the OMM in WT and KO conditions. Only when there is a change in Fis1’s level on the OMM, can the authors claim that Yta4 removes Fis1 to regulate mito fission.

We are pleased that the reviewer was convinced that Fis1 is a Yta4 client protein when Yta4 is overexpressed. In addition, we would like to point out that the physical interaction between Yta4 and Fis1 is unambiguous in the present study (Figs 6C, 8C, and S5B).

We agree with the reviewer that the claim that Yta4 removes Fis1 to regulate mitochondrial fission can be made only when there is a change in the level of Fis1 on the OMM in cells lacking Yta4. To test this in a quantitative manner, we followed the suggestion to quantify the average intensity of GFP-Fis1 signals on mitochondria in WT and *yta4*∆ cells (see new S3E Fig). The result showed that GFP-Fis1 signals were comparable in WT and *yta4*∆ cells, suggesting that Yta4 may play a minor role in extracting Fis1, at least under physiological conditions. Therefore, we have carefully revised text, as well as the proposed model in Fig 12, to demonstrate that Fis1 delocalization is caused by Yta4 overexpression. Nonetheless, we would like to provide a few points below to explain why it is challenging to observe alteration of Fis1 on mitochondria in cells lacking Yta4.

1) Yta4 is a versatile protein and has many client proteins, including the divisome components Fis1, Mdv1, and Dnm1 discovered in the present study. Therefore, under physiological conditions, the percentage of Yta4 molecules that interact with Fis1 may be limited.

2) Yta4 may form hexamers or monomers within the cell. The mechanisms underlying the recognition of client proteins by Yta4/Msp1 remain unclear. Multiple models have been proposed (Wang and Walter, 2020). In one of the models, Monomeric Msp1 (a homolog of Yta4 in budding yeast) recruits client proteins before forming hexamers. Fis1 is a typical TA protein and requires Yta4 to form hexamers for extraction. Therefore, the possible existence of multiple forms of Yta4 within the cell may decrease the frequency of Yta4 in extracting Fis1.

3) Fis1 is a resident TA protein on the OMM. Unlike TA proteins mistargeted to mitochondria, Fis1 is destined for the OMM once born. This means that Fis1 is present on the OMM regardless of the presence of Yta4. In this scenario, it is more sensitive to detect the presence of mistargeted TA proteins, but not the alteration of the resident TA protein Fis1, on the OMM in cells lacking Yta4 as there is no such mistargeted TA proteins on the OMM in cells containing Yta4.

Given the three points listed above, overexpression of Yta4 is expected to have a significant effect on the localization of Fis1 to mitochondria as the presence of more Yta4 molecules is expected to increase the chance of the interaction between Fis1 and Yta4 and the number of Yta4 hexamers.

Finally, to reflect our observations and thoughts, the statements below were added to the Result and Discussion sections in the revised manuscript:

1) Statements in the Result section

“*In contrast to the significant effect of Yta4 overexpression on Fis1, the absence of Yta4 did not appear to significantly alter the mitochondrial localization of Fis1 (Fig 4C and 4E). Consistently, quantification showed that the average intensity of GFP-Fis1 signals on mitochondria was not significantly altered by the absence of Yta4 (S3E Fig). These results indicate that under physiological conditions, Yta4 may play a minor role in extracting Fis1 from the mitochondrial outer membrane. However, it is also possible that under physiological conditions, Yta4-mediated extraction of Fis1 takes place less frequently at only a few subregions on mitochondria, which may not be discernible (see discussion for details)* ”

2) Statements in the Discussion section

*“However, we noticed that the absence of Yta4 did not significantly alter the localization of Fis1 on mitochondria (Figs 4E and S3E). Considering that Yta4 have many client proteins, including Mdv1 and Dnm1, on the mitochondrial outer membrane, we speculate that the minor effect of the absence of Yta4 on Fis1 could be due to the infrequent extraction of Fis1 by Yta4 under physiological conditions. In addition, within the cell, Yta4 may not be present as only hexamers, a form required for extracting TA proteins.”*

For the second claim, the authors showed the Yta4 inhibits oligomer formation of Dnm1, since that is presented as a substantial piece of data in the paper, the authors should clarify whether the inhibition of Dnm1-Mdv1 interaction observed is due to the inhibition of oligomerization of Dnm1. That is, whether oligomerization of Dnm1 is required for the Dnm1-Mdv1 interaction.

To address this concern, an assembly-defective mutant of Dnm1, i.e., Dnm1(G380D) (Dnm1(G385D) in budding yeast), was used. It has been reported that the budding yeast Dnm1(G385D) is defective in oligomerization/assembly (Ingerman et al., 2005; Lackner et al., 2009).

1) We first confirmed the defective assembly of the fission yeast Dnm1(G380D) by size-exclusion chromatography (S6 Fig), as reported previously (Ingerman et al., 2005). The elution volume of Dnm1(G380D) increased significantly, suggesting that Dnm1(G380D) is defective in assembly.
2) By performing careful GST pull-down assays (four independent experiments), we tested the interaction of GST-Mdv1(WD) with Dnm1(WT) and Dnm1(G380D) (lanes 2 and 5 in S6B Fig). The results showed that the interaction of GST-Mdv1(WD) with Dnm1(WT) and Dnm1(G380D) was comparable (lanes 2 and 5 in S6B and S6C Fig), suggesting that Dnm1 oligomerization is not required for the Dnm1-Mdv1 interaction.
3) We further tested the effect of Yta4 on the interaction of GST-Mdv1(WD) with Dnm1(WT) and Dnm1(G380D) (lanes 3 and 6 in S6B and S6C Fig). Consistent with the data shown in Figs 9A and 9C, Yta4 inhibited the interaction of GST-Mdv1(WD) with Dnm1(WT) and Dnm1(G380D) in a comparable manner.

Collectively, the results suggest that Dnm1 oligomerization is not required for the Dnm1-Mdv1 interaction. The following statements were added to the Result section:

“*Considering that Yta4 also inhibits Dnm1 oligomerization (see below in Fig 11), we tested whether the inhibitory effect of Yta4 on the interaction between Dnm1 and Mdv1 (Fig 9A and 9B) was due to the inhibition of Dnm1 oligomerization. We used an assembly-defective mutant of Dnm1 (i.e., Dnm1(G385D)), which was reported previously [37, 38]. Sequence alignment exhibited that the Glycine residue at 385 in the Saccharomyces cerevisiae Dnm1 is conserved in the Schizosaccharomyces pombe Dnm1 (i.e., Glycine at 380) (S6A Fig). Therefore, to generate the Schizosaccharomyces pombe version of a Dnm1 assembly-defective mutant, we mutated the Glycine residue at 380 in Dnm1 to Aspartic acid (referred to as Dnm1(G380D)). Consistent with the previous finding [37], size exclusion chromatography confirmed that Dnm1(G380D) was defective in assembly (S6A Fig). Using Dnm1(G380D) and its wild-type version (referred to as Dnm1(WT)), we performed similar competitive binding assays, as shown in Fig 9A. As showed in S6B and S6C Fig, Dnm1(WT) and Dnm1(G380D) interacted with GST-Mdv1(WD) in a comparable manner, and His-Yta4(∆TM) inhibited the interaction of GST-Mdv1(WD) with Dnm1(WT) and Dnm1(G380D) in a comparable manner. Thus, we concluded that the inhibitory effect of Yta4 on the interaction between Dnm1 and Mdv1 is not due to the inhibition of Dnm1 oligomerization.”*

For the third claim, I am convinced that Yta4 inhibits the GTPase activity. But again, how is this related to the inhibition of oligomerization? Since the authors presented a three-fold mechanism here, they should make sure that the three reasons are orthogonal to each other. The role of Dnm1 oligomerization in yta4’s inhibition to its activity needs to be fleshed out.

To address this concern, we similarly used the assembly-defective mutant Dnm1(G380D), indicated above.

In fact, it is technically challenging to clearly demonstrate how Yta4 inhibits the GTPase activity because of the complex and intertwining effects of Dnm1, listed below as reported previously (Ingerman et al., 2005; Lackner et al., 2009).

1) GTP drives Dnm1 assembly.
2) Dnm1 assembly in turn promotes GTP hydrolysis.
3) Dnm1 spontaneously self-assembles to form heterogeneous oligomers in vitro.

Therefore, to reduce the complexity of analyzing how Yta4 regulates the GTPase activity of Dnm1. Oligomerization of Dnm1 was avoided by using the assembly-defective mutant Dnm1(G380D) in colorimetric assays, which were used to determine the GTPase kinetics of Dnm1. Intriguingly, Dnm1(G380D) still displayed a GTPase activity but with a very low rate of GTP hydrolysis (S8 Fig) and the presence of His-Yta4(∆TM) significantly increased the K_m_ value, but not the V_max_ value, of Dnm1(G380D). This result unambiguously suggests that Yta4 can inhibit the GTPase activity of Dnm1 independent of Dnm1 oligomerization.

The statements below were added to the Result section:

“*GTP drives Dnm1 assembly, and Dnm1 assembly in turn stimulates GTP hydrolysis of Dnm1 [37, 38]. Moreover, Dnm1 can self-assemble to form heterogenous oligomers in vitro [37, 38]. These intertwining effects of Dnm1 make it challenging to clearly determine how Yta4 affects the GTPase activity of Dnm1. To reduce the complexity in analyzing Dnm1 GTPase activity, we performed colorimetric assays by using the assembly-defective version of Dnm1 (i.e., Dnm1(G380D), see S6A Fig). Interestingly, Dnm1(G380D) still exhibited a GTPase activity but has a very low rate of GTP hydrolysis (Km=85.00 µM, Vmax=1.02 µM/min, vs the control Dnm1(WT): Km=126.59 µM, Vmax=4.82 µM/min) (S8 Fig). Of note, the presence of His-Yta4(∆TM) similarly increased the Km values, but not the Vmax values, of both Dnm1(WT) and Dnm1(G380D) (S8 Fig). This result suggests that Yta4 can inhibit the GTPase activity of Dnm1 independent of Dnm1 self-assembly.”*

Overall, I recommend more experiments and text revision to flesh out the model before acceptance of publication.

We hope these additional rigorous experiments would satisfactorily address the concerns.

**References:**

Ingerman, E., E.M. Perkins, M. Marino, J.A. Mears, J.M. McCaffery, J.E. Hinshaw, and J. Nunnari. 2005. Dnm1 forms spirals that are structurally tailored to fit mitochondria. *J Cell Biol*. 170:1021-1027.

Lackner, L.L., J.S. Horner, and J. Nunnari. 2009. Mechanistic analysis of a dynamin effector. *Science*. 325:874-877.

Wang, L., and P. Walter. 2020. Msp1/ATAD1 in Protein Quality Control and Regulation of Synaptic Activities. *Annu Rev Cell Dev Biol*. 36:141-164.
